# Buwang Formula Regulates Microglial Metabolic Reprogramming and Modulates the mTOR/HIF-1α Pathway to Reduce Neuroinflammation in Diabetic Mice

**DOI:** 10.3390/ph19071032

**Published:** 2026-07-01

**Authors:** Tong Su, Yinian Men, Xiaochen Li, Lingling Qin, Lili Wu, Tonghua Liu

**Affiliations:** 1 The Second Clinical Medical College, Beijing University of Chinese Medicine, Beijing 100078, China; 20230941274@bucm.edu.cn (T.S.); 20240941296@bucm.edu.cn (Y.M.); 2 Key Laboratory of Health Cultivation of the Ministry of Education, Beijing University of Chinese Medicine, Beijing 102488, Chinaqingniao_566@163.com (L.W.); 3 School of Traditional Chinese Medicine, Beijing University of Chinese Medicine, Beijing 102488, China

**Keywords:** Buwang formula, diabetes-associated cognitive dysfunction, metabolic reprogramming, microglia, mTOR/HIF-1α pathway, neuroinflammation

## Abstract

**Background**: Microglial metabolic reprogramming drives neuroinflammation in Diabetes-associated cognitive dysfunction (DACD). This study aims to evaluate Buwang formula (BWF) effects on diabetic neuroinflammation and microglial metabolism. **Methods**: The chemical constituents present in BWF-containing cerebrospinal fluid (BWF-CCSF) were profiled by UHPLC-MS/MS, and putative targets were predicted via network pharmacology analysis. Diabetic db/db mice were treated with BWF, and behavioral, biochemical, and histopathological assessments were performed. The in vivo findings were further validated in BV2 cells exposed to high glucose (HG) and palmitic acid (PA). Cellular energy metabolism analysis was used to quantify dynamic changes in oxidative phosphorylation (OXPHOS) and glycolysis in BV2 cells, while flow cytometry and immunofluorescence were used to examine BV2 cell polarization. The expression levels of pathway-related proteins were examined by Western blot analysis. **Results**: A total of 15 chemical components were identified in BWF-CCSF. According to the network pharmacology prediction, the mTOR/HIF-1α pathway might participate in the effects exerted by BWF compounds that enter the brain. In diabetic mice, BWF notably suppressed the expression of pro-inflammatory factors and reduced the accumulation of pathological proteins within the hippocampal tissue, which improved learning and memory impairments, and these improvements were accompanied by suppressed activation of the mTOR/HIF-1α pathway and its downstream glycolysis. In BV2 cells exposed to HG and PA, BWF-CCSF treatment significantly increased OXPHOS and inhibited glycolysis, promoting a polarization toward M2 anti-inflammatory phenotype. **Conclusions**: BWF regulates microglial metabolic reprogramming and attenuates neuroinflammation, effects that are associated with modulation of the mTOR/HIF-1α pathway, and these findings suggest that BWF warrants further investigation as a potential therapeutic candidate for DACD.

## 1. Introduction

Diabetes mellitus (DM) is frequently accompanied by cognitive impairment, a common neurological complication, known as diabetes-associated cognitive dysfunction (DACD), which affects up to 40.8% of patients [1]. This condition involves deficits in multiple cognitive domains and may progress to deterioration of daily living abilities and personality changes in severe cases [2]. DACD contributes to reduced self-management capacity, increased dependence on nursing care, and impaired glycemic control, thereby severely compromising patients’ quality of life and overall health. Currently, no standardized pharmacological treatment for DACD has been established. Clinical management primarily relies on glucose-lowering agents with neuroprotective potential [3,4], or on symptomatic therapies adapted from dementia treatment guidelines [5]. Nevertheless, drugs specifically indicated for DACD remain lacking, and interventions capable of delaying or reversing disease progression are still limited. This highlights the pressing demand for novel therapeutic options specifically designed for the precise clinical management of DACD.

Accounting for approximately 5–12% of the brain’s total cellular content, microglia constitute the primary innate immune sentinels within the central nervous system and play a fundamental role in controlling the onset, progression, and termination of inflammatory responses in neural tissue [6]. Under the diabetic pathological milieu, metabolic stressors such as sustained hyperglycemia, insulin resistance, and dysregulated lipid metabolism [7] can drive microglia to shift from a resting metabolic state predominantly reliant on oxidative phosphorylation (OXPHOS) toward a pro-inflammatory glycolytic phenotype, a process termed metabolic reprogramming [8,9,10,11]. This metabolic transition promotes microglial polarization toward a pro-inflammatory phenotype, accompanied by the secretion of abundant inflammatory mediators [9], disruption of synaptic function, and suppression of neuroprotective functions, ultimately culminating in neuronal injury and cognitive decline [12,13]. A key pathological mechanism underlying this shift is the aberrant activation of the mammalian target of rapamycin (mTOR)/hypoxia-inducible factor-1α (HIF-1α) pathway, which acts as a critical metabolic sensor [14]. Hyperactivation of this axis robustly drives the transcription and expression of glycolytic genes, thereby supplying the necessary energy and biosynthetic building blocks for microglia to adopt a pro-inflammatory M1 phenotype [15,16,17]. Consequently, targeting the mTOR/HIF-1α pathway to modulate microglial metabolic reprogramming and induce a shift toward the M2 anti-inflammatory state has been increasingly recognized as a potential therapeutic approach [8]. For instance, Katarina et al. [16] demonstrated that agmatine suppressed the phosphatidylinositol 3-hydroxy kinase (PI3K)/protein kinase B (AKT)/mTOR/HIF-1α cascade in lipopolysaccharide-stimulated BV2 cells, thereby attenuating glycolysis, reducing lactate and tumor necrosis factor α (TNFα) production, and preserving mitochondrial integrity while inhibiting apoptosis. A microglia-targeted nanomodulator designed for immunometabolic reprogramming was recently reported, which [18], by inhibiting the AKT/mTOR/HIF-1α pathway, induced a metabolic shift away from glycolysis toward OXPHOS in immune-stimulated microglia and drove their conversion from a pro-inflammatory M1 to an anti-inflammatory M2 phenotype, ultimately mitigating neuroinflammation and neurodegenerative pathology in mice with Alzheimer’s disease (AD).

Buwang Formula (BWF) is a traditional Chinese herbal prescription composed of five medicinal materials: Ginseng Radix, Polygalae Radix, Acori Tatarinowii Rhizoma, Poria, and Poria cum Radix Pini. It has been used clinically for the treatment of dementia for centuries. Current pharmacological evidence indicates that the bioactive constituents derived from these herbs, such as Ginsenoside Rg1 [19,20], Asarone [21], Senegenin [22], and Poria cocos polysaccharide [23,24], exert anti-neuroinflammatory effects and improve cognitive function. Nevertheless, whether these constituents can directly traverse the blood–brain barrier (BBB) and the extent to which they synergistically modulate microglial metabolic reprogramming following brain entry remain incompletely elucidated.

This study integrated the identification of BBB-penetrating constituents, network pharmacology prediction, and cellular energy metabolism phenotyping. First, the active constituents of BWF capable of traversing the BBB were identified, and their potential targets and pathways were explored using network pharmacology. Subsequently, the functional effects and underlying mechanisms were evaluated by combining in vivo and in vitro approaches. This study aims to provide a novel mechanistic interpretation and experimental foundation for the therapeutic application of BWF in DACD.

## 2. Results

### 2.1. Identification of Compounds in BWF Aqueous Extract, BWF-Containing Serum and BWF-Containing Cerebrospinal Fluid

**Figure 1:**
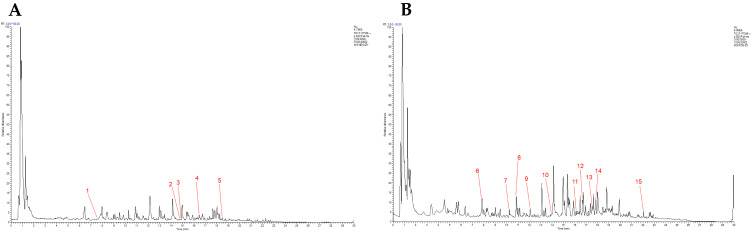
Identification of compounds in BWF aqueous extract and BWF-CCSF. (**A**) The components in the BWF aqueous extract detected in negative ion mode. The red markers represented the components that were subsequently detected in the BWF-CCSF. (**B**) The components in the BWF aqueous extract detected in positive ion mode. The red markers represented the components that were subsequently detected in the BWF-CCSF. (1) Chlorogenic acid. (2) Ginsenoside Re. (3) Ginsenoside Rg1. (4) Polygalasaponin XXIV. (5) Ginsenoside Rd. (6) Sinapic Acid. (7) Sibiricaxanthone B. (8) Polygalaxanthone XI. (9) Cimifugin. (10) 5,7,4′-Trihydroxy-6-methoxyflavanone. (11) 3″O-Acetylplatycodin D. (12) 26-Hydroxyporicoic acid DM. (13) Ganolucidic acid B. (14) Ginsenoside Rb2. (15) Poricoic acid B.

Ultra-high performance liquid chromatography-tandem mass spectrometry (UHPLC-MS/MS) analysis identified 199 chemical components in BWF aqueous extract. A total of 103 components in BWF-containing serum and 15 brain-permeable components in BWF-containing cerebrospinal fluid (BWF-CCSF) were identified (Figure 1A,B); the detailed detection data of BWF-CCSF are provided in Table 1. The components in BWF-CCSF were mainly classified into triterpenoids, terpene glycosides, and benzopyrans, comprising 11 parent compounds and four phase II metabolites. Based on fragment ion information, the four metabolites were identified as glucuronidated products of the parent compounds from Polygalae Radix, Acori Tatarinowii Rhizoma, Poria cum Radix Pini, and Poria, respectively. The Supplementary Figure S1 shows the extraction ion current diagrams of 15 components in BWF-CCSF, and the Supplementary Tables S1–S3 provide the detailed information on the detected compounds in BWF aqueous extract, BWF-containing serum, and BWF-CCSF.

### 2.2. Identification of Shared Drug–Disease Targets

**Figure 2:**
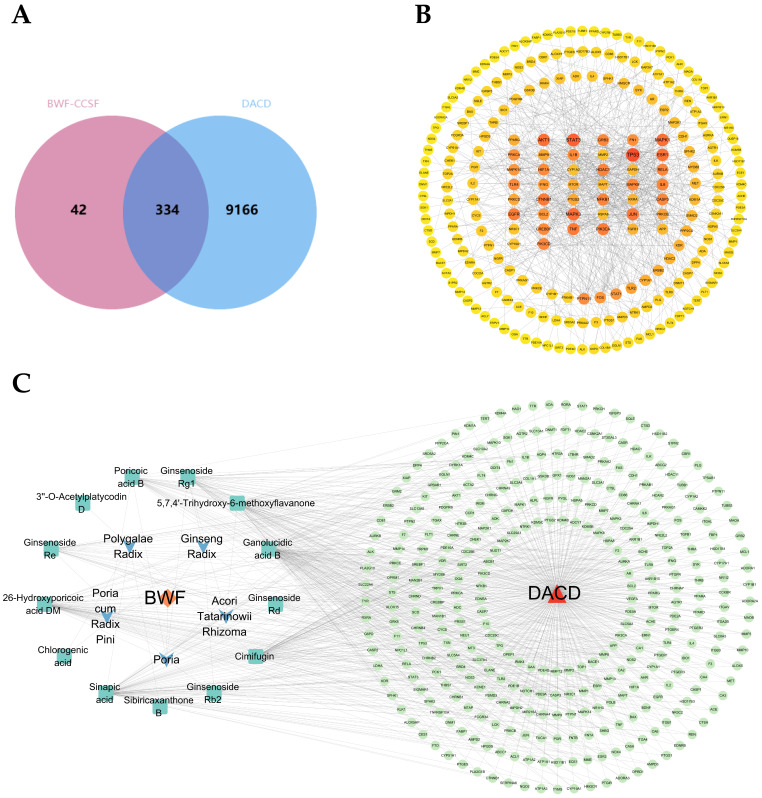
Screening of shared drug–disease targets and generation of the interaction network. (**A**) A Venn intersection analysis diagram of the shared predicted targets of BWF-CCSF and DACD. (**B**) The PPI network of the shared predicted targets. The larger the node and the redder its color, the more central it is in the PPI network; the targets arranged in a matrix form were the hub genes. (**C**) A visualization of herb–component–target–disease network. Nodes of different colors and shapes represented different components in the network, and the lines connecting them represented the predicted potential relationships.

A total of 9500 DACD-related targets were retrieved from the GeneCards database. The predicted targets of 13 components identified in BWF-CCSF (the remaining two components yielded no predicted targets) were integrated and deduplicated, yielding 376 unique targets. The intersection of the two sets was analyzed and visualized, resulting in 334 shared drug–disease targets (Figure 2A). These targets were then employed to generate the herb–component–target–disease network and to perform functional enrichment analysis.

### 2.3. Protein–Protein Interaction Analysis and Identification of Hub Targets

The 334 overlapping targets were uploaded to the STRING database to build a protein–protein interaction (PPI) network representing BWF brain-permeable components that directly intervene in DACD. A total of 331 nodes were included in the analysis, comprising 829 known or predicted interactions. The median Degree and Betweenness centrality values of the network were 6.05 and 635.65, respectively. Nodes with both values above the respective medians were identified as hub genes, yielding 49 hub genes (Figure 2B).

### 2.4. Visualization of the Herb–Component–Target–Disease Network

An herb–component–target–disease network was generated and visualized (Figure 2C), illustrating the potential associations among five herbs, 15 active components, and 334 targets. Based on topological analysis, with the median Degree of the network used as the threshold, seven core components were identified: Ganolucidic acid B, Sinapic acid, Cimifugin, 5,7,4′-Trihydroxy-6-methoxyflavanone, 26-Hydroxyporicoic acid DM, Ginsenoside Rg1, and Poricoic acid B.

### 2.5. Enrichment Analyses

**Figure 3:**
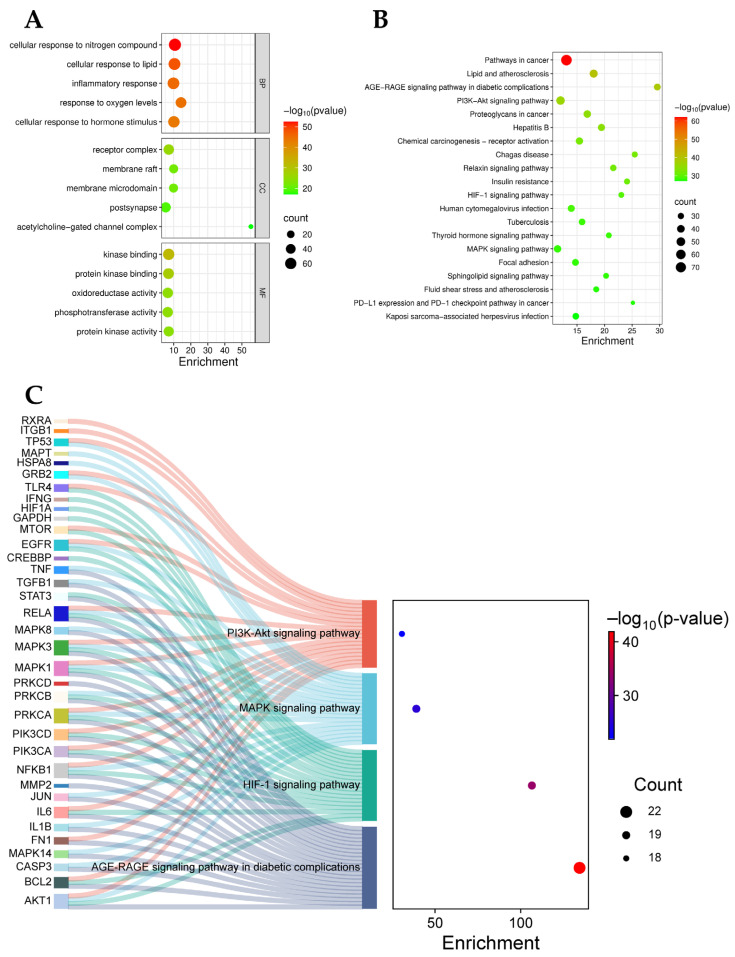
Functional enrichment analyses of shared drug–disease targets and hub genes. (**A**) GO-based functional annotation of the shared predicted targets of BWF-CCSF and DACD. (**B**) KEGG-based functional annotation of the shared predicted targets of BWF-CCSF and DACD. (**C**) The KEGG enrichment of hub genes in the key metabolic pathways related to DACD. In the bubble chart, the horizontal axis represented the enrichment factor, the size of the bubbles indicated the number of differentially expressed genes enriched in that term, and the color represented the −log10 *p*-value, which is the significance of the enrichment. Abbreviations: BP, biological process; CC, cellular component; MF, molecular function.

The overlapping targets between the drug and the disease were subjected to functional enrichment analysis using the Gene Ontology (GO) database and the Kyoto Encyclopedia of Genes and Genomes (KEGG) pathway resource. Ranked by log_10_ *p*-value in ascending order, the top five enriched GO terms (biological process, cellular component, molecular function) and the top 20 KEGG pathways were displayed (Figure 3A,B). GO analysis revealed that the target genes were predominantly enriched in responses to various stimuli, including lipids, inflammation, hypoxia, and hormones, and in the regulation of the mitogen-activated protein kinase (MAPK) cascade. These genes were mainly localized to receptor complexes, membrane microdomains, and postsynaptic components, and their molecular functions involved oxidoreductase, protein kinase, and phosphotransferase activities, as well as nuclear receptor and transcription factor regulation.

Representative enriched KEGG pathways included the advanced glycation end-products (AGEs)–receptor for advanced glycation end-products (RAGE) pathway, PI3K/AKT pathway, HIF-1 pathway, and MAPK pathway. The associations between core target genes and these pathways were further visualized (Figure 3C).

### 2.6. BWF Improves Fasting Blood Glucose and Lipid Metabolism Profile in Diabetic Mice

**Figure 4:**
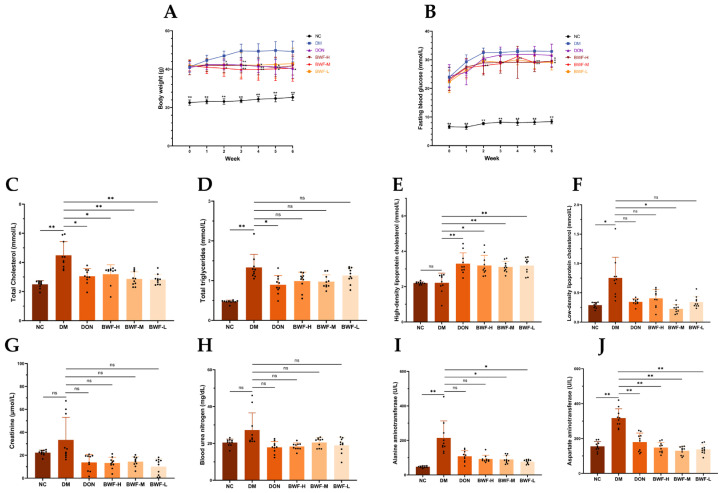
Assessment of BWF on glucolipid metabolism and organ function in diabetic mice. (**A**) Dynamic curve of body weight of mice (*n* = 10). (**B**) Dynamic curve of FBG of mice (*n* = 10). (**C**–**F**) Effect of BWF intervention on the blood lipid profiles of diabetic mice (*n* = 10). (**G**,**H**) Assessment of renal function after BWF intervention (*n* = 10). (**I**,**J**) Assessment of hepatic function after BWF intervention (*n* = 10). Comparisons with the DM group: ** *p* < 0.01; * *p* < 0.05; ns, not significant.

Throughout the experimental period, body weight and fasting blood glucose (FBG) were measured once a week. At baseline, there were no significant differences in FBG or body weight across the db/db groups. After the intervention, body weight gain in all BWF dose groups slowed, and during the week 2 to week 5, the body weight of the donepezil (DON), BWF high-dose (BWF-H) and BWF medium-dose (BWF-M) groups showed markedly reduced levels compared with the DM group (Figure 4A). Furthermore, from week 4 onward, FBG in the BWF low-dose (BWF-L) group, BWF-M group, BWF-H group decreased sequentially relative to the DM group (Figure 4B). Analysis of circulating lipids (Figure 4C–F) revealed that, relative to the DM group, total cholesterol (TC) in serum was reduced and high-density lipoprotein cholesterol (HDL-C) was elevated across all BWF-treated groups. Triglycerides (TG) concentrations remained comparable among the groups, whereas low-density lipoprotein cholesterol (LDL-C) showed a notable decrease solely in the BWF-M group. Hepatic and renal safety parameters were also assessed. Renal function markers, including serum creatinine (CREA) and blood urea nitrogen (BUN), were comparable across all groups (Figure 4G,H), indicating that BWF administration did not impose additional renal stress on diabetic mice. In contrast, circulating alanine aminotransferase (ALT) and aspartate aminotransferase (AST) concentrations were markedly lower in the BWF-treated groups than in the DM group (Figure 4I,J), pointing to a possible hepatoprotective role of BWF.

### 2.7. BWF Treatment Preserves Cognitive Function in Diabetic Mice

**Figure 5:**
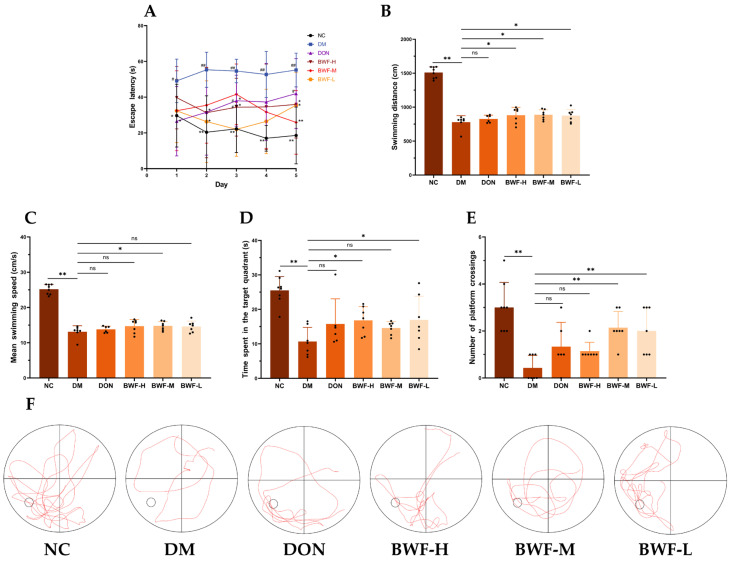
Effects of BWF on learning and memory impairment in diabetic mice. (**A**) The escape latency of mice in the place navigation test (*n* = 6–8). (**B**–**E**) The swimming distance, mean swimming speed, time spent in the target quadrant, and number of platform crossings of mice in the spatial probe trial (*n* = 6–8). (**F**) Typical swimming trajectories of all groups in the spatial probe trial (*n* = 6–8). Comparisons with the NC group: ## *p* < 0.01; # *p* < 0.05; comparisons with the DM group: ** *p* < 0.01; * *p* < 0.05; ns, not significant.

In the place navigation test, the escape latency of mice in the normal control (NC) group exhibited an overall decreasing trend across the training days. The DM group exhibited significantly longer escape latencies relative to the NC group across the testing days. On the final day, escape latency in all BWF dose groups was significantly shortened (Figure 5A). A spatial probe trial was conducted to assess memory function. Compared with the DM group, mice in the BWF dose groups showed varying degrees of increase in total swimming distance, mean swimming speed, time spent in the target quadrant, and number of platform crossings (Figure 5B–E). Figure 5F presents typical swimming trajectories of all groups in the spatial probe trial.

### 2.8. Effect of BWF on Pathological Morphology of Hippocampus in Diabetic Mice

**Figure 6:**
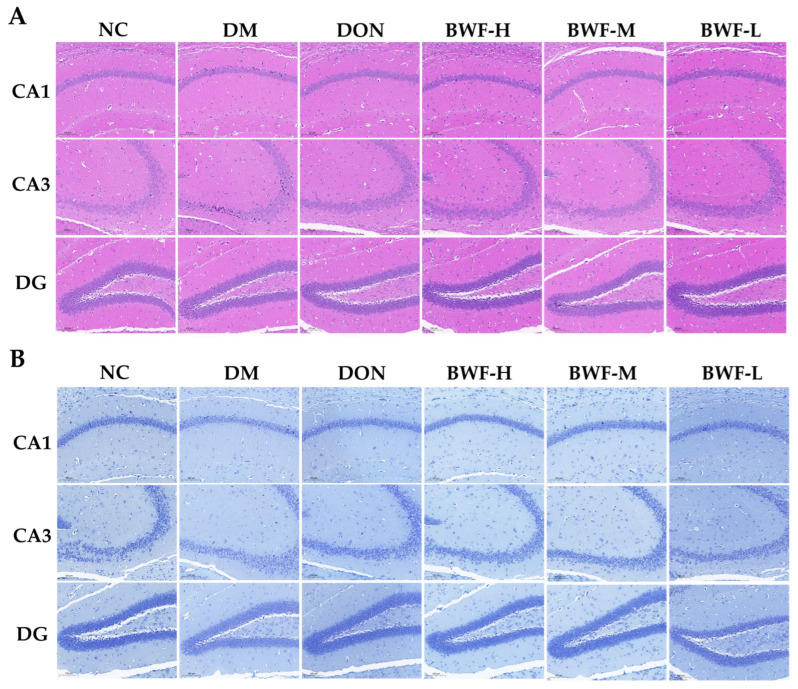
Hippocampus morphology and histopathological staining. (**A**) Representative H&E micrographs (scale bar = 100 μm; original magnification, 400×; *n* = 3). (**B**) Representative Nissl micrographs (scale bar = 100 μm; original magnification, 400×; *n* = 3).

Hematoxylin and eosin (H&E) staining of the hippocampus showed that neurons in the NC group were abundant, tightly arranged, and free of degeneration, necrosis, or structural disorganization. In the DM group, the neuronal arrangement was slightly disordered, intercellular spaces were moderately widened, and neuronal loss was evident, with scattered nuclear pyknosis. In the DON group and BWF dose groups, neuronal arrangement remained mildly irregular, but nuclear pyknosis was less pronounced (Figure 6A). Nissl staining revealed abundant Nissl bodies in the neuronal cytoplasm of mice in the NC group, appearing as dark blue granules or clumps with uniform distribution. In the DM group, Nissl bodies were markedly reduced or absent, and cytoplasmic staining was pale. The hippocampal Nissl body content in the DON and all BWF-treated groups was notably elevated relative to that of the DM group (Figure 6B).

### 2.9. BWF Alleviated Neuroinflammation and Pathological Protein Accumulation in the Hippocampus of Diabetic Mice

**Figure 7:**
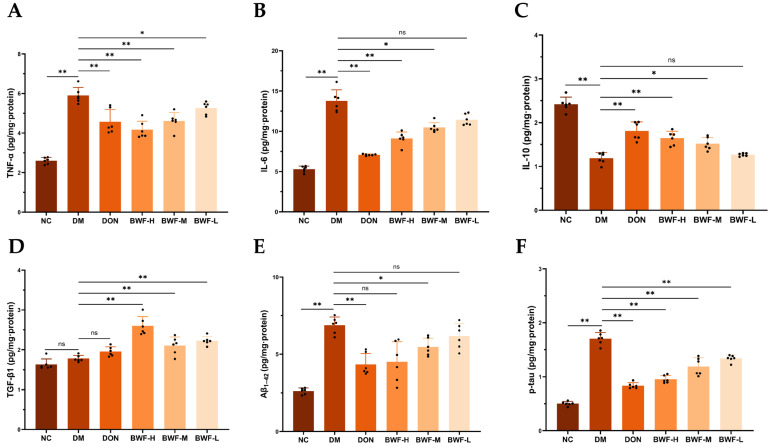
BWF alleviated hippocampal inflammation and reduced pathological protein accumulation in diabetic mice. (**A**–**D**) Hippocampal concentrations of TNF-α, IL-6, IL-10 and TGF-β1 (*n* = 6). (**E**,**F**) Hippocampal concentrations of Aβ_1–42_ and p-tau (*n* = 6). Comparisons with the DM group: ** *p* < 0.01; * *p* < 0.05; ns, not significant.

Inflammatory cytokine measurements revealed marked neuroinflammation in the hippocampus of db/db mice. Relative to the DM group, BWF treatment reduced hippocampal TNF-α and interleukin-6 (IL-6) concentrations, while elevating those of IL-10 and transforming growth factor-β1 (TGF-β1) (Figure 7A–D). Furthermore, the levels of the pathological proteins β-amyloid_1–42_ (Aβ_1–42_) and p-tau (Thr181) were elevated within the DM group, while both were decreased following BWF intervention (Figure 7E,F).

### 2.10. BWF-Treated Diabetic Mice Showed Reduced Hippocampal mTOR/HIF-1α Pathway Activation and Glycolysis

**Figure 8:**
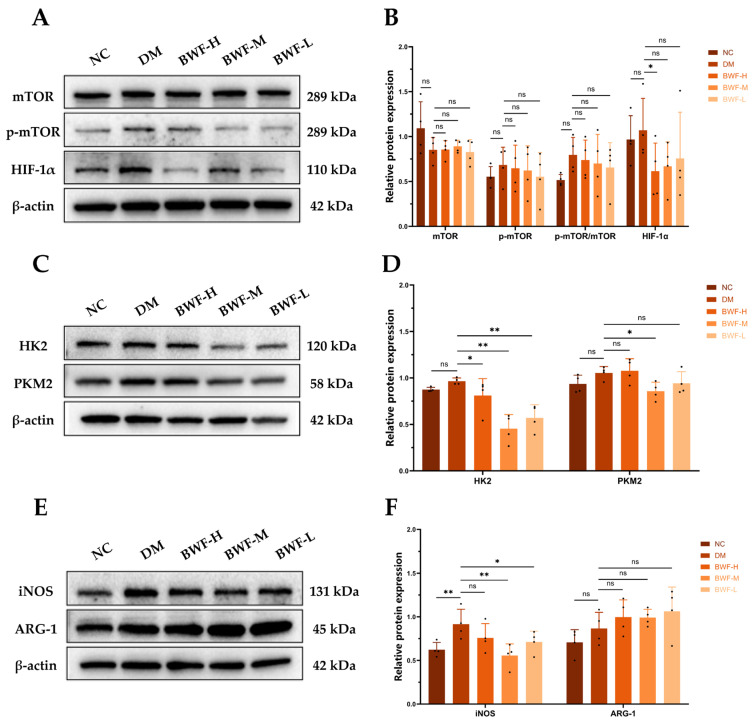
Regulation of mTOR/HIF-1α pathway-related and glycolytic proteins by BWF in the diabetic mouse hippocampus. (**A**,**B**) mTOR, p-mTOR and HIF-1α protein expression levels (*n* = 4). (**C**,**D**) HK2 and PKM2 protein expression levels (*n* = 4). (**E**,**F**) iNOS and ARG-1 protein expression levels (*n* = 4). Comparisons with the DM group: ** *p* < 0.01; * *p* < 0.05; ns, not significant.

Based on the network pharmacology results, the predicted targets of brain-permeable BWF constituents showed significant enrichment in metabolic pathways associated with mTOR and HIF-1α. To further corroborate these results, hippocampal tissues from all groups were analyzed for protein levels linked to the mTOR/HIF-1α signaling axis (Figure 8A). Relative to the DM group, the p-mTOR/mTOR ratio showed a slight decrease in all BWF dose groups but was not significantly different, whereas HIF-1α levels were markedly decreased in the BWF-H group (Figure 8B). Protein levels of hexokinase 2 (HK2), a critical glycolytic enzyme, were markedly reduced across all BWF-treated groups, whereas pyruvate kinase M2 (PKM2) was significantly lowered in the BWF-M group (Figure 8C,D). These results indicate that BWF may suppress the activity of glycolytic enzymes acting downstream of HIF-1α. With respect to polarization markers, the M1-associated inducible nitric oxide synthase (iNOS) was markedly lower in the NC, BWF-M, and BWF-L groups relative to the DM group, while the M2 marker arginase-1 (ARG-1) exhibited an upward trend across all BWF intervention groups, although this trend did not attain statistical significance (Figure 8E,F).

### 2.11. BWF-CCSF Regulated DM-Induced Polarization of BV2 Cells to M2 Phenotype

**Figure 9:**
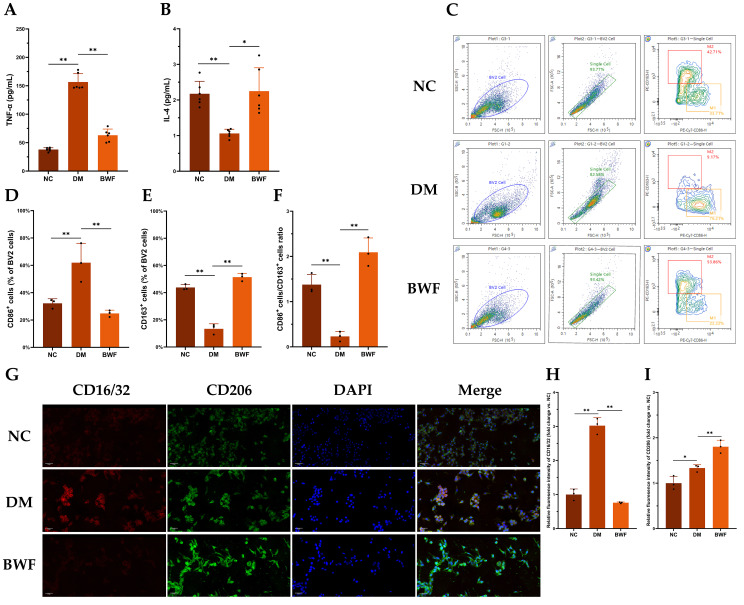
Effects of BWF-CCSF on the polarization phenotype in the DM-induced BV2 cell model. (**A**,**B**) Concentrations of TNF-α and IL-4 in BV2 cell culture supernatants (*n* = 6). (**C**) Flow cytometry assessment of CD86 and CD163 in BV2 cells (*n* = 3). (**D**–**F**) The proportion of CD86^+^ and CD163^+^ cells and the ratio of CD163^+^/CD86^+^ analyzed by flow cytometry (*n* = 3). (**G**) Representative immunofluorescence plots of BV2 cells stained for CD16/32 and CD206 (scale bar = 50 μm; original magnification, 400×; *n* = 3). (**H**,**I**) Normalized mean fluorescence intensity of CD16/32 and CD206 in BV2 cells (*n* = 3). Comparisons with the DM group: ** *p* < 0.01; * *p* < 0.05.

Compared with the DM group, BWF-CCSF intervention significantly increased the IL-4 level and markedly decreased the TNF-α level in the BV2 culture supernatant (Figure 9A,B). Flow cytometry provided further supporting evidence (Figure 9C): the proportion of CD86^+^ cells, an M1 polarization marker, was markedly reduced in the BWF group compared with the DM group, while the proportion of CD163^+^ cells, an M2 marker, and the M2/M1 ratio were significantly higher (Figure 9D–F). Consistent results were obtained by immunofluorescence staining using CD16/32 (M1) and CD206 (M2) (Figure 9G): CD16/32 expression decreased while CD206 expression increased in the BWF group (Figure 9H,I). Collectively, these findings suggest that BWF-CCSF promotes the polarization of DM-induced BV2 cells from a pro-inflammatory M1 to an anti-inflammatory M2 phenotype.

### 2.12. BWF-CCSF Inhibited DM-Induced Glycolysis and Promoted the Recovery of OXPHOS in BV2 Cells

**Figure 10:**
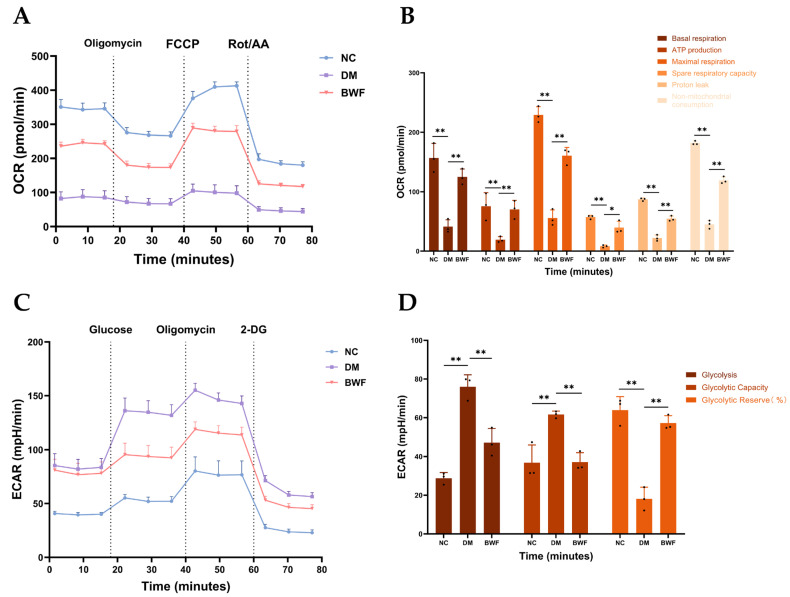
Effects of BWF-CCSF on the glycolysis and OXPHOS in the DM-induced BV2 cell model. (**A**) The dynamic curve of OCR of BV2 cells in mitochondrial stress test (*n* = 3). (**B**) The 6 characteristics related to OCR in mitochondrial stress test (*n* = 3). (**C**) The dynamic curve of ECAR of BV2 cells in glycolysis stress test (*n* = 3). (**D**) The 3 characteristics related to ECAR in glycolysis stress test (*n* = 3). Comparisons with the DM group: ** *p* < 0.01; * *p* < 0.05.

The mitochondrial stress test revealed that the overall oxygen consumption rate (OCR) profile was reduced in the DM group relative to the NC group. Specifically, all measured parameters were significantly decreased, including basal respiration, maximal respiration, ATP production, spare respiratory capacity, non-mitochondrial oxygen consumption, and proton leak (Figure 10A,B), indicating impaired mitochondrial OXPHOS and reduced stress reserve capacity in BV2 cells under DM conditions. After BWF-CCSF intervention, these parameters significantly recovered, suggesting restoration of OXPHOS function.

The glycolysis stress test indicated that the extracellular acidification rate (ECAR) was markedly higher in the DM group relative to the NC group, accompanied by an increase in basal glycolysis, while glycolytic reserve capacity was reduced, indicating a shift in energy production toward glycolysis in BV2 cells under DM conditions, with glycolytic flux approaching its maximal output. Following BWF-CCSF intervention, basal glycolysis decreased relative to the DM group, glycolytic reserve capacity recovered, and glycolytic capacity did not differ significantly from the NC group (Figure 10C,D). These findings indicate that BWF-CCSF can suppress the overactivation of glycolysis under DM conditions while preserving the glycolytic stress reserve capacity of the cells.

### 2.13. BWF-CCSF Treatment Was Associated with Suppressed mTOR/HIF-1α Pathway-Mediated Metabolic Reprogramming in DM-Induced BV2 Cells

**Figure 11:**
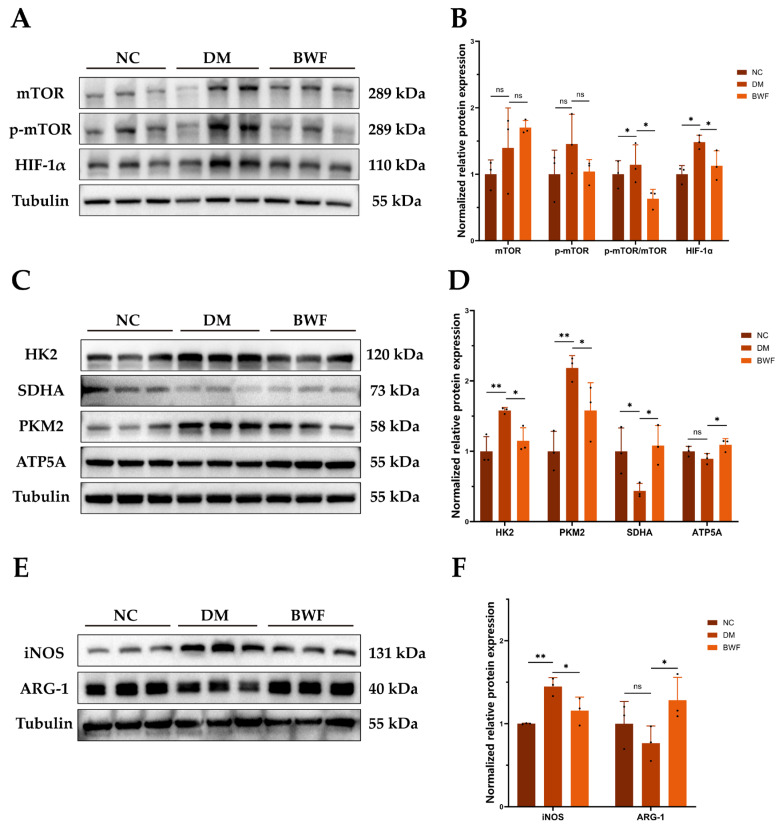
BWF-CCSF regulated the expression of proteins associated with mTOR/HIF-1α-driven metabolic reprogramming in BV2 cells. (**A**,**B**) mTOR, p-mTOR and HIF-1α normalized relative protein levels (*n* = 3). (**C**,**D**) HK2, SDHA, PKM2 and ATP5A normalized relative protein levels (*n* = 3). (**E**,**F**) iNOS and ARG-1 normalized relative protein levels (*n* = 3). Comparisons with the DM group: ** *p* < 0.01; * *p* < 0.05; ns, not significant.

Consistent with the in vivo results yet exhibiting greater clarity, the BV2 cell experiments showed that BWF-CCSF exerted a more pronounced regulatory influence on the mTOR/HIF-1α signaling axis and its downstream glycolytic targets. The p-mTOR/mTOR ratio and the protein levels of HIF-1α, HK2, PKM2, and iNOS were markedly reduced in the BWF group compared with the DM group (Figure 11A,B). Notably, the OXPHOS-related proteins Succinate dehydrogenase [ubiquinone] flavoprotein subunit, mitochondrial (SDHA) and ATP synthase subunit alpha, mitochondrial (ATP5A) (Figure 11C,D), as well as the M2 marker ARG-1 (Figure 11E,F), were significantly upregulated.

## 3. Discussion

DM is widely recognized as a predisposing condition for mild cognitive decline and dementia [25]. As the global demographic shifts toward an older population, the incidence of DACD continues to rise each year, posing a growing public health challenge [26]. Neuroinflammation serves as a key link between DM and cognitive impairment [27]. Essentially a defense mechanism, a moderate inflammatory response facilitates tissue repair and the clearance of harmful substances. However, when inflammation persists and acute inflammation transitions to chronic inflammation, the pro-inflammatory response continuously outweighs the anti-inflammatory response. The sustained release of inflammatory cytokines aggravates neuronal damage and eventually leads to cognitive dysfunction [27].

Neuroinflammation is critically driven by metabolic reprogramming occurring in microglia. Evidence indicates that pro-inflammatory (M1) microglia rely primarily on glycolysis to satisfy their high energy requirements, whereas anti-inflammatory/reparative (M2) microglia are more dependent on OXPHOS and fatty acid oxidation [28]. Accordingly, reshaping this metabolic imbalance through pharmacological intervention has emerged as a novel strategy for treating neurodegenerative diseases [29]. However, the contribution of microglial metabolic reprogramming to DACD has not been fully characterized, and no drug targeting neuroinflammation has been widely approved for clinical use. In this context, adjunctive therapies, particularly traditional Chinese medicine (TCM), have gained considerable interest because of their ability to act through multiple targets and signaling pathways simultaneously. UHPLC-MS/MS was employed in the current study to comprehensively characterize the components present in the aqueous extract of BWF, which is traditionally used to treat dementia, and in its corresponding drug-containing cerebrospinal fluid (BWF-CCSF). Network pharmacology analysis was then employed to predict potential pathways, and the results converged on the mTOR/HIF-1α pathway, which is related to microglial metabolic reprogramming. These findings underwent subsequent experimental validation.

Before discussing the pharmacological mechanisms, it is important to clarify the dose selection of BWF and its relationship to official standards. It should be noted that BWF is not listed as a proprietary preparation in the current edition of the *Chinese Pharmacopeia*, but is a classical prescription derived from *Beiji Qianjin Yaofang*, a seminal classical text of TCM. The closely related formula Kai-Xin-San, which shares four of the five herbs in BWF, is officially documented in the *Catalog of Ancient Classic Famous Formulas (First Batch)* [30]. A recent 2025 study also reported the same formula under the name Bu-Wang-San, with an identical five-herb composition and inter-herb dose ratios [31]. The human therapeutic daily dose of BWF was derived from this classical text, and the animal doses and in vitro concentration used in this study were calculated from this clinical baseline. The daily clinical doses were: Ginseng Radix 15 g (*Chinese Pharmacopeia* recommended: 3–9 g) [32], Acori Tatarinowii Rhizoma 6 g (*Chinese Pharmacopeia* recommended: 3–10 g) [32], Poria 15 g (*Chinese Pharmacopeia* recommended: 10–15 g) [32], Polygalae Radix 21 g (*Chinese Pharmacopeia* recommended: 3–10 g) [32], and Poria cum Radix Pini 15 g. Accordingly, the human therapeutic daily dose of BWF was 72 g. For Poria cum Radix Pini, which is not an independent monograph in the *Chinese Pharmacopeia*, its dose was cross-referenced with provincial standards, which consistently specify a maximum dose of 15 g: the *Hubei Standard (2018)* [33], the *Shaanxi Standard (2015)* [34], and the *Jiangsu Standard (2020)* [35]. The doses of Ginseng Radix and Polygalae Radix exceed the general *Chinese Pharmacopeia* ranges; this reflects the clinical practice in classical TCM formulas, where higher doses are frequently prescribed for severe conditions such as dementia. The animal doses (0.5-fold, 1-fold, and 2-fold) were calculated from this baseline via body surface area conversion, and the 10% BWF-CCSF concentration corresponded to the effective in vivo dose. These dose equivalences support the translational relevance of the findings. In terms of safety, a recent pharmacological study of Bu-Wang-San reported a rat dose exceeding our highest mouse dose [31], and an acute toxicity study of the closely related formula Kai-Xin-San documented an oral median lethal dose (LD_50_) of approximately 32.59 g/kg in mice [36], which is more than ten times higher than the doses used in our experiments, further supporting the safety profile of this formula family at the employed dosage range.

Our study indicated that in the hippocampus of DACD mice, microglia are excessively polarized toward pro-inflammatory M1 phenotype, while anti-inflammatory M2 phenotype is relatively insufficient, resulting in a marked pro-inflammatory/anti-inflammatory imbalance. This imbalance was reflected by markedly upregulated TNF-α and IL-6 levels, along with downregulated IL-10 and TGF-β1 levels. In addition, hippocampal pathological proteins Aβ_1–42_ and p-tau levels were significantly increased [37], closely mirroring the AD-like pathological features. All of these pathological changes were substantially ameliorated following BWF intervention. These in vivo improvements in neuroinflammation and pathological protein deposition are consistent with the restoration of microglial metabolic homeostasis observed in our in vitro experiments, suggesting that BWF may alleviate DACD pathology by rebalancing microglial glycolysis and OXPHOS. Building on these observations, we subsequently explored the signaling cascades potentially responsible for the benefits conferred by BWF. Accumulating evidence has established that anti-inflammatory cytokines, most notably TGF-β1 and IL-10, are critically involved in the removal of disease-associated proteins. In particular, TGF-β1 can engage the mothers against decapentaplegic homolog 3 (SMAD3) signaling cascade within microglia, resulting in the upregulation of scavenger receptors and a marked enhancement of the cellular ability to engulf Aβ and p-tau [38,39]. Concurrently, TGF-β1 can suppress glycogen synthase kinase-3 beta (GSK-3β) via the PI3K/AKT signaling cascade, reducing the excessive phosphorylation of tau protein at its source [35]. Thus, BWF may facilitate the shift of microglia toward an M2 anti-inflammatory state and enhance the secretion of anti-inflammatory mediators, especially TGF-β1, thereby activating microglial phagocytic function and inhibiting tau phosphorylation. This cascade ultimately reduces the hippocampal pathological deposition of Aβ and p-tau and ameliorates the learning and memory deficits in diabetic mice. It is also worth considering that BWF improved systemic metabolic parameters, including FBG and serum lipid profiles. Therefore, the cognitive benefits observed in vivo may partially reflect indirect effects of ameliorated glucolipotoxicity rather than exclusively direct central mechanisms. Nonetheless, our in vitro findings confirm that BWF-CCSF can directly modulate microglial metabolic reprogramming, suggesting that systemic effects and direct central actions likely work in concert to produce the overall therapeutic benefits of BWF.

In vitro experiments demonstrated that the DM microenvironment established by high glucose combined with palmitic acid led to a comprehensive impairment of mitochondrial respiratory function in BV2 cells. All measured OCR-related parameters were significantly decreased in the DM group, which was consistent with observations in certain in vitro high-glucose models. Chen et al. [40] reported that high glucose did not significantly alter basal respiration in human brain microvascular endothelial cells but markedly decreased maximal respiration and spare respiratory capacity, with proton leak also showing a declining trend; Zhang et al. [41] isolated cardiac microvascular endothelial cells from DM mice and likewise found significant decreases in non-mitochondrial oxygen consumption and proton leak. Our data showed a marked decrease in the absolute proton leak level within the DM group, which may be related to the overall downregulation of mitochondrial respiratory activity under high glucose, leading to a decrease in total proton efflux. BWF-CCSF intervention significantly restored these parameters, indicating that BWF redirects metabolic flux toward OXPHOS, thereby providing the bioenergetic foundation required for maintaining the M2 anti-inflammatory phenotype of microglia. Meanwhile, glycolysis stress test results showed that basal glycolysis was significantly elevated in DM-stimulated BV2 cells while the glycolytic reserve capacity was reduced, indicating a shift in energy production toward glycolysis, with the glycolytic flux approaching its maximal output. BWF-CCSF intervention not only returned the aberrantly elevated basal glycolysis to normal but also effectively reestablished the glycolytic reserve capacity, enabling cells to retain the ability for rapid energy production under stress. This dual regulation, which suppresses pathological glycolytic overflow while preserving adaptive reserve, may help prevent the chronic glycolytic dependence that drives M1 polarization, while preserving the ability of microglia to execute appropriate inflammatory responses aimed at repairing damaged tissue and clearing noxious agents [42,43].

mTOR constitutes a pivotal downstream node of PI3K/AKT signaling and a crucial upstream trigger of HIF-1α [44]. Network pharmacology analysis revealed that the predicted targets of brain-permeable BWF components were significantly enriched in mTOR/HIF-1α-related pathways, a result corroborated by our in vivo and in vitro experimental findings. DM-related metabolic stresses, such as hyperglycemia and AGEs [45,46], can aberrantly activate HIF-1α even under normoxic conditions. This process is thought to be driven by the buildup of tricarboxylic acid cycle intermediates, notably succinate [47], which inhibits prolyl hydroxylase activity and prevents the ubiquitination and degradation of HIF-1α, allowing it to accumulate stably in the nucleus [8,48]. As a transcription factor, HIF-1α then directly upregulates a range of glycolytic genes, such as lactate dehydrogenase A (LDHA), HK2, PKM2, and pyruvate dehydrogenase kinase (PDK) [49]. Among these, LDHA converts pyruvate into lactate, and PDK1 inhibits the pyruvate dehydrogenase complex, preventing pyruvate from entering the mitochondria for oxidative metabolism; together, they reinforce glycolytic flux. HK2 functions as a critical rate-limiting enzyme in microglial glycolysis and is recruited to mitochondria under inflammatory activation to promote glycolytic flux and inflammatory processes. Indeed, it has been shown that downregulation of HK2 can alleviate microglia-mediated neuroinflammation [50]. PKM2, another essential glycolytic rate-limiting enzyme, contributes to various pathological processes in the nervous system, including inflammatory responses, oxidative damage, and programmed cell death [51,52]. The activities of both HK2 and PKM2 are under the transcriptional control of HIF-1α. Cell-based experiments have indicated that when HIF-1α is overexpressed in microglia, the levels of PKM2 and HK2 rise, along with enhanced secretion of IL-6, IL-17, and TNF-α [53]. Conversely, when HIF-1α is inhibited by sirtuin-1 (SIRT1) in microglia, the activities of the glycolytic-related GLUT1, HK2, PGK1, and LDH are reduced, and iNOS, IL-6, and TNF-α levels are significantly decreased, suppressing the inflammatory process [54]. These lines of evidence indicate that HIF-1α promotes the release of inflammatory molecules by regulating glycolysis, and that inhibiting glycolysis may produce similar anti-inflammatory and neuroprotective effects [55]. Glycolysis inhibitors such as 2-DG or HK2 inhibitors can directly target the core of microglial energy metabolism, showing substantial promise for treating various neurodegenerative diseases, including AD [49,56]. In our study, BWF-CCSF treatment was associated with attenuated mTOR/HIF-1α signaling activation, along with decreased expression of its downstream glycolytic targets. These changes may contribute to the rebalancing of microglial energy metabolism away from pro-inflammatory glycolysis.

In addition to suppressing glycolysis, BWF significantly upregulated the OXPHOS-related SDHA and ATP5A levels. SDHA is the core catalytic subunit of mitochondrial respiratory chain complex II, responsible for oxidizing succinate to fumarate and transferring electrons to ubiquinone, thereby connecting the tricarboxylic acid cycle with the mitochondrial electron transport chain [57]. Its downregulation impedes the conversion of succinate to fumarate, potentially leading to reduced tricarboxylic acid cycle flux and abnormal succinate accumulation. This has been shown to inhibit prolyl hydroxylase activity, thus stabilizing HIF-1α and further driving the metabolic shift of macrophages toward glycolysis [47]. ATP5A is the catalytic core subunit of the F1 domain of ATP synthase, and its downregulation directly impairs ATP synthesis, resulting in insufficient cellular energy supply and promoting the transition of microglia to an M1 phenotype, together with the stimulation of inflammatory signaling [58]. Therefore, the upregulation of SDHA by BWF may decrease succinate buildup, leading to enhanced HIF-1α degradation, while the restoration of ATP5A may ensure adequate ATP supply for maintaining the M2 phenotype. Together, these effects reinforce the shift from glycolysis to OXPHOS and help resolve neuroinflammation. BWF-CCSF restored OXPHOS function by increasing the expression of mitochondrial respiratory chain-related proteins in BV2 cells, indicating its potential as a dual regulator of glycolysis and OXPHOS.

It is worth noting that the Western blot analysis of hippocampal tissue revealed that certain proteins, such as the p-mTOR/mTOR ratio and ARG-1, did not reach statistical significance, although trends toward decreased mTOR phosphorylation and increased ARG-1 expression were observed in the BWF-treated groups. This lack of statistical significance may be attributable to the cellular heterogeneity of the hippocampus, which contains not only microglia but also neurons, astrocytes, and oligodendrocytes. The total tissue lysate, therefore, reflects an averaged signal across all cell types. As noted in recent studies, bulk tissue analyses average signals across multiple cell types, which can obscure functional heterogeneity and fail to capture rare cell-type-specific signatures [59,60,61]. In contrast, the in vitro experiments using BV2 cells, which avoid this dilution effect, clearly demonstrated that BWF-CCSF significantly suppressed the mTOR/HIF-1α pathway and downstream glycolytic enzymes while upregulating OXPHOS-related proteins. Taken together, the in vivo protein expression data show directional trends consistent with the in vitro findings but should be regarded as supporting rather than independently confirmatory evidence for the proposed mechanism. These complementary findings point to the possibility that the regulatory effect of BWF on microglial metabolic reprogramming is cell-type specific and support the necessity of using purified microglia or single-cell approaches in future in vivo studies to capture such changes with greater sensitivity.

This study has several limitations. First, the chemical components identified in the BWF aqueous extract and BWF-CCSF were not precisely quantified, and no pharmacokinetic studies were conducted. Additionally, only a single batch of BWF aqueous extract was used in this study; therefore, batch-to-batch consistency was not assessed. Subsequent work should focus on these issues to promote the standardization of BWF preparation and administration, and to establish more refined dosage ranges to determine its optimal therapeutic window. Second, although we anticipate that multiple components in BWF may act synergistically on multiple targets to treat DACD, the specific contribution of each component to the regulation of microglial metabolic reprogramming could not be determined in this study. Third, it should also be acknowledged that BV2 cells are an immortalized line and do not entirely recapitulate the phenotypic and functional characteristics of primary microglia in vivo. Finally, although the present work measured protein levels within the mTOR/HIF-1α signaling pathway, functional verification of key targets using inhibitor interventions or gene knockdown/knockout approaches was not performed. Hence, the current work should be recognized as establishing an association rather than a direct causal relationship between BWF-mediated mTOR/HIF-1α modulation and microglial metabolic reprogramming. However, our multi-level evidence, which includes network pharmacology predictions and in vivo and in vitro protein expression data, provides a solid foundation for this mechanistic hypothesis and identifies specific directions for future functional validation. Given these limitations, the present findings support an association between BWF treatment and modulation of the mTOR/HIF-1α signaling pathway in microglia, but the precise causal relationship and clinical translation require further pharmacokinetic characterization and functional validation.

## 4. Materials and Methods

### 4.1. Preparation of BWF Aqueous Extract

The composition and individual doses of BWF were as described in the discussion. To meet the dosage requirements of both the in vivo and in vitro experiments, the herbs were proportionally scaled up from the human therapeutic daily dose. The final amounts used for extraction were Ginseng Radix 1200 g, Acori Tatarinowii Rhizoma 480 g, Poria 1200 g, Poria cum Radix Pini 1200 g, and Polygalae Radix 1680 g. All herbs were purchased from Tongrentang Co., Ltd., Beijing, China. The medicinal materials were mixed, washed, and soaked in pure water at a material-to-solvent volume ratio of 1:10. The mixture was soaked for 30 min and then boiled for 1.5 h. The first extract was collected by filtration through a strainer, and the residue was extracted a second time following the same procedure. The two extracts were pooled and evaporated under vacuum, and then freeze-dried under vacuum to obtain 858 g of BWF aqueous extract powder, corresponding to an extraction yield of 14.9%. The powder was sealed and stored at −20 °C. All herbal raw materials used in this study were obtained from a single production batch, and the BWF aqueous extract was prepared by a qualified company (Zhenweikang Biotechnology Co., Ltd., Nanjing, China) using a mature extraction process to ensure consistency.

### 4.2. Preparation of BWF-Containing Serum and BWF-CCSF

The preparation of BWF-containing serum and BWF-CCSF was adapted and improved from the protocol reported by Wu et al. [62]. Male SD rats (8 weeks old, 300–400 g) were purchased from Yaokang Biotechnology Co., Ltd., Beijing, China and housed under specific-pathogen-free (SPF) conditions (temperature: 22 ± 2 °C, relative humidity: 50% ± 5%, 12 h light/dark cycle) with a quiet environment and were allowed unrestricted access to food and water. Following a one-week acclimatization period, rats were allocated to a control group (*n* = 30) and a treatment group (*n* = 15) based on body weight. To meet the requirements of ingredient identification, the rat dose was set at twice the human clinical dose. According to the clinical daily dose used in humans (72 g/70 kg/day), the body surface area conversion factor (6.3), and the extraction yield (14.9%), the rat dose was calculated as 1.93 g/kg/day. The BWF aqueous extract was suspended in deionized water and orally gavaged once a day for five consecutive days, whereas control animals received an equal volume of deionized water. Two hours following the final dose, anesthesia was induced by intraperitoneal administration of 2.5% tribromoethanol (IA296R, Solomon Biotechnology, Tianjin, China). After deep anesthesia was confirmed, abdominal aortic blood was drawn, and rats were secured in a brain stereotaxic device; the foramen magnum was exposed, cerebrospinal fluid (CSF) was collected from the cisterna magna using a microsyringe, and then the rats were subsequently euthanized. The collected CSF and clotted whole blood were centrifuged, and the resulting supernatants were heat-treated at 56 °C for 30 min and then filtered through a 0.22 μm membrane to remove any microbial contamination. Filtrates from the same group were combined, aliquoted into sterile cryotubes, and kept at −80 °C. The resulting preparations were designated as blank CSF, BWF-CCSF, blank serum and BWF-containing serum.

### 4.3. UHPLC-MS/MS Analysis of BWF Aqueous Extract and BWF-CCSF

One gram of BWF aqueous extract powder was accurately weighed, and 20 mL of 80% methanol (A456-4, Thermo Fisher Scientific, Waltham, MA, USA) was added [63,64]. This solvent was selected as a well-established compromise solvent that enables simultaneous extraction of both highly polar and less polar constituents, which is essential for comprehensive chemical profiling of traditional Chinese medicine formulas. The mixture was sonicated for 1 h. Following centrifugation, the supernatant was mixed with an equal volume of ultrapure water (W6-4, Thermo Fisher Scientific, Waltham, MA, USA) and transferred to an autosampler vial. For serum and CSF samples, 100 µL of each sample was mixed with 300 µL of methanol, vortexed, and centrifuged under the same conditions. The supernatant was concentrated by vacuum centrifugation, reconstituted in 50% aqueous methanol, and centrifuged again before UHPLC-MS/MS analysis. Chromatographic separation was achieved using a column (2.1 mm × 100 mm, 1.8 μm, Waters Corporation, Milford, MA, USA). The mobile phase consisted of 0.1% formic acid (A117-50, Thermo Fisher Scientific, Waltham, MA, USA) in water (A) and acetonitrile (B) (A955-4, Thermo Fisher Scientific, Waltham, MA, USA). The gradient was programmed from 98% A to 70% A over 14 min, and then to 0% A at 25 min, before re-equilibration. The separation was conducted at 40 °C, at a flow of 0.3 mL/min, with a 6 μL injection. Mass spectrometric detection was carried out using electrospray ionization (ESI) in both positive and negative modes, under the source settings described by Liu et al. [64]. Mass spectrometric data were analyzed with Progenesis QI (v3.0, Waters Corporation, Milford, MA, USA), and compound annotation relied on retention time, fragment ion matching, isotopic distribution, precursor ion mass accuracy, and peak intensity.

### 4.4. Network Pharmacology

#### 4.4.1. Target Prediction of Brain-Permeable BWF Components

Since the blood–brain barrier keeps most substances out of the brain, the components identified in the BWF-CCSF represent the pool of candidates capable of directly acting on brain cells. Structural information for these active components was obtained from PubChem (https://pubchem.ncbi.nlm.nih.gov/, accessed on 22 March 2025). The components were then submitted to the online platforms SwissTargetPrediction (https://www.swisstargetprediction.ch/, accessed on 22 March 2025), BATMAN (http://bionet.ncpsb.org.cn/batman-tcm/, accessed on 22 March 2025), and HERB (http://47.92.70.12/, accessed on 22 March 2025). The predictions obtained from different databases were then integrated, and duplicates were removed to yield the predicted brain targets of BWF. Human target genes associated with DACD were retrieved from the GeneCards database (https://www.genecards.org/, accessed on 22 March 2025). The DACD-related targets and the predicted targets of the brain-permeable BWF components were subjected to Venn analysis using Venny (https://bioinfogp.cnb.csic.es/tools/venny/, accessed on 22 March 2025). The intersection was considered the shared drug–disease targets and was defined as the potential target genes for the direct intervention and treatment of DACD by BWF in the brain.

#### 4.4.2. PPI Analysis and Screening of Hub Genes

A PPI network was built by inputting the shared drug–disease targets into the STRING database (https://cn.string-db.org/, accessed on 25 March 2025) with the confidence threshold set at 0.9. After removing disconnected nodes, network visualization and analysis were performed using Cytoscape (v3.10.2, https://cytoscape.org/, accessed on 25 March 2025). Degree and Betweenness centrality were calculated, and the median value of each parameter was used as the threshold. Nodes with both values above the thresholds were identified as hub target genes.

#### 4.4.3. Generation of the Herb–Component–Target–Disease Network

Based on the shared drug–disease targets, the component–target correspondence was mapped. The resulting data were loaded into Cytoscape to build the herb–component–target–disease network for visualization of the interactions.

#### 4.4.4. Functional Enrichment Analyses

GO and KEGG enrichment analyses were performed on Metascape (https://metascape.org/, accessed on 25 March 2025). GO enrichment analysis covered three categories: BP, CC, and MF. To further focus on the metabolic pathways closely related to the pathogenesis of DACD, the associations between hub target genes and the enriched pathways were visualized.

### 4.5. Animals and Treatment

A total of 50 SPF male db/db mice with spontaneous DM (11 weeks old, 36–45 g) were used as the DACD animal model, and 10 wild-type (wt/wt) mice served as the NC group. We acquired all mice from Beijing Yaokang Biotechnology Co., Ltd., Beijing, China and housed them under SPF conditions in a quiet environment. Except for a 6 h fasting period before FBG measurement, the mice had unrestricted access to food and water. Following one week of acclimation, FBG was measured in the 50 db/db mice via tail vein blood sampling. DM was defined as an FBG level higher than 11.1 mmol/L. Subsequently, the db/db mice were assigned to five experimental groups (*n* = 10/group) by stratified randomization based on FBG and body weight: DM group, BWF-H group, BWF-M group, BWF-L group, and DON group as the positive control.

Mice in the BWF-H, BWF-M, and BWF-L groups received BWF aqueous extract by gavage at doses of 2.8 g/kg/day, 1.4 g/kg/day, and 0.7 g/kg/day, respectively. These doses were calculated based on body surface area conversion (human-to-mouse conversion factor: 9.1) and the extraction yield (14.9%) of the BWF aqueous extract, corresponding to 2-fold, 1-fold, and 0.5-fold of the clinical dose, respectively. The DON group received donepezil (Aricept, 5 mg, Eisai, Tokyo, Japan) at 0.65 mg/kg/day by gavage. The selection of donepezil as the positive control was based on the evidence that it exerts neuroprotective effects in T2DM animals by ameliorating AD-like pathological changes and has been widely used as a comparator in studies on DACD [65,66]. All drugs were dissolved in deionized water; the NC and DM groups were given deionized water via gavage. The intervention lasted for 6 consecutive weeks. During the experiment, FBG was measured weekly via tail vein blood sampling, and fasting body weight was recorded.

### 4.6. Morris Water Maze Test

After 6 weeks of BWF intervention, cognitive function was assessed with the Morris water maze test, which used a round pool (120 cm in diameter, 50 cm in depth) containing water made opaque and maintained at 22 ± 1 °C. In the adaptive training session, a platform was positioned 1 cm above the water surface in one quadrant. Mice were released from the opposite quadrant and allowed to swim for 60 s; those failing to locate the platform were guided to it and left there for 15 s [67]. Animals with visual or motor impairments, or those unable to identify or stay on the platform, were excluded during this phase. The place navigation test was performed over five consecutive days, with the platform hidden 1 cm below the water surface, and escape latency (maximum 60 s) was recorded. One day after, spatial memory was assessed in a probe test with the platform taken away, and the time spent in the target quadrant, number of platform crossings, total swimming distance, and mean swimming speed were quantified to evaluate spatial memory.

### 4.7. Tissue Collection and Processing

After a 6-week intervention, anesthesia (R510-22-10, RWD Life Science, Shenzhen, China) was induced with isoflurane in all mice. Once deep anesthesia was established, retro-orbital blood was quickly drawn, and the mice were immediately sacrificed via cervical dislocation. Residual blood was removed by transcardial perfusion with saline, after which the whole brains were rapidly excised on ice. Three brains per group were randomly selected and fixed in 4% paraformaldehyde (P1110, Solarbio Science & Technology, Beijing, China); the hippocampal tissues from the remaining brains were quickly dissected on ice, quickly frozen in liquid nitrogen and kept at −80 °C. The collected blood samples were clotted and then centrifuged, and the serum supernatant was carefully harvested and kept at −80 °C.

### 4.8. Biochemical and Molecular Analysis

Circulating levels of TC, TG, HDL-C, and LDL-C were determined with an automatic biochemical analyzer (BS-420, Mindray Bio-Medical Electronics, Shenzhen, China) to evaluate the effects of BWF on blood lipid profiles. Serum CREA, BUN, ALT, and AST were also determined to assess the hepatic and renal safety of BWF. The levels of TNF-α (HJ207, Epizyme Biomedical Technology, Shanghai, China), IL-6 (HJ182, Epizyme Biomedical Technology, Shanghai, China), IL-10 (HJ185, Epizyme Biomedical Technology, Shanghai, China), TGF-β1 (HJ205, Epizyme Biomedical Technology, Shanghai, China), Aβ_1–42_ (HY-D0068, Huaying Biotechnology, Beijing, China), and p-tau (Thr181) (HY-NE458, Huaying Biotechnology, Beijing, China) in mouse hippocampal homogenates were determined using enzyme-linked immunosorbent assay (ELISA) kits following the manufacturer’s guidelines.

### 4.9. Histological Staining

Hippocampal morphology was evaluated using hematoxylin–eosin (H&E) pathological staining. After overnight fixation in 4% paraformaldehyde, brains were sectioned coronally to expose the hippocampal region. Coronal brain slices (approximately 3 mm thick) were dehydrated, paraffin-embedded and cut into 4 μm sections. Paraffin sections were dewaxed in xylene (10023418, Sinopharm Chemical Reagent, Shanghai, China), rehydrated in a descending ethanol (100092683, Sinopharm Chemical Reagent, Shanghai, China) series, and then stained with hematoxylin following the standard H&E staining protocol (BH0001, Powerful Biotechnology, Wuhan, China). After differentiation in hydrochloric acid and bluing in ammonia water, the sections were counterstained with eosin. Once staining was complete, the sections were dehydrated through ascending concentrations of ethanol, cleared using xylene, and sealed with neutral balsam before being examined and photographed under a microscope (ECLIPSE CI, Nikon Corporation, Tokyo, Japan).

Nissl staining was performed to observe morphological changes of neurons in the mouse hippocampal region. Paraffin sections were heated at 60 °C for 30 min, dewaxed using xylene, passed through a descending ethanol series for rehydration, and washed with distilled water. The sections were then immersed in Nissl staining solution (BH0013, Powerful Biotechnology, Wuhan, China) and stained at 60 °C for 10 min, followed by a gentle rinse in distilled water to remove excess stain. Differentiation was carried out in glacial acetic acid (G10000218, Sinopharm Chemical Reagent, Shanghai, China) until the background was clear and Nissl bodies appeared dark blue, after which distilled water washing was used to stop differentiation. Subsequent processing steps were carried out in the same manner as the H&E staining procedure described above.

### 4.10. Cell Culture, Modeling and Treatment

BV2 cells (YC-C035, Yuanjing Biotechnology, Guangzhou, China), a mouse microglial line, were cultured in DMEM (11995065, Gibco, Waltham, MA, USA) enriched with 10% fetal bovine serum (FBS) (10091148, Gibco, Waltham, MA, USA) and 1% penicillin–streptomycin (15070063, Gibco, Waltham, MA, USA). Cultures were incubated at 37 °C in a humidified environment with 5% CO_2_. When the cells reached 80–90% confluence under microscopic observation, they were digested, prepared as a single-cell suspension, and seeded into well plates for subsequent experiments.

The experimental setup consisted of three groups: NC group, DM group, and BWF group. Each group was seeded into three independent wells on separate plates (*n* = 3/group). After the cells entered the logarithmic growth phase, the NC group was treated with normal culture medium containing 10% blank CSF. An in vitro DM model was established using high glucose (HG, 100 mM) (SL6450, Coolaber Science & Technology, Beijing, China) combined with palmitate (PA, 100 μM) (IPA13840, Solarbio Science & Technology, Beijing, China). A CCK-8 assay identified 10% BWF-CCSF as the optimal non-cytotoxic concentration for BV2 cell treatment, based on a gradient of tested doses (Figure S2). On this modeling basis, the DM group and the BWF group were treated with medium containing 10% blank CSF and 10% BWF-CCSF, respectively, for 24 h.

### 4.11. Collection and Detection of Culture Supernatant

In 12-well plates, BV2 cells were plated at 2 × 10^5^ per well. Once the 24 h treatment was complete, we removed the culture medium and washed the cells with PBS (P1020, Solarbio Science & Technology, Beijing, China). After replacing with fresh complete medium, the cells were cultured for another 12 h, and the resulting conditioned medium was collected, centrifuged, and filtered through a 0.22 µm membrane. The concentrations of TNF-α and IL-4 (HJ179, Epizyme Biomedical Technology, Shanghai, China) in the conditioned medium were measured by ELISA.

### 4.12. Flow Cytometry

BV2 cells were seeded in 12-well plates and treated for 24 h as described above; then, the cells were harvested and stained with fluorophore-conjugated antibodies against CD86 (105013, BioLegend, San Diego, CA, USA) and CD163 (105013, BioLegend, San Diego, CA, USA) for 30 min in the dark at 4 °C. Following incubation, cells were rinsed and suspended in staining buffer (C1711, Beyotime Biotechnology, Shanghai, China). Data were acquired on an LSRFortessa flow cytometer (LSRFortessa, BD Biosciences, San Jose, CA, USA), and at least 1 × 10^4^ events were recorded per sample. The percentages of CD86^+^ and CD163^+^ cells were analyzed with FACSDiva software (v9.0, BD Biosciences, San Jose, CA, USA), and the M2/M1 ratio (CD163^+^/CD86^+^) was calculated to assess BV2 cell polarization.

### 4.13. Cellular Energy Metabolism Analysis

BV2 cells were plated in a Seahorse XF96 assay plate (Agilent Technologies, Santa Clara, CA, USA) at 1 × 10^4^ cells/well and cultured overnight to allow attachment. After the 24 h treatment described above, the medium was replaced with assay medium, and the cells were equilibrated in a CO_2_-free incubator for 1 h. The mitochondrial stress test (103015-100, Agilent Technologies, Santa Clara, CA, USA) and glycolysis stress test (103020-100, Agilent Technologies, Santa Clara, CA, USA) were carried out on a Seahorse XFe96 Analyzer (Agilent Technologies, Santa Clara, CA, USA) following the manufacturer’s protocols [68]. For the mitochondrial stress test, oligomycin (1.5 μM), FCCP (1 μM), and rotenone/antimycin A (0.5 μM) were added sequentially. For the glycolysis stress test, glucose (10 mM), oligomycin (1 μM), and 2-DG (50 mM) were injected in sequence. Three measurement cycles were recorded after each injection, and OCR and ECAR were monitored in real time.

### 4.14. Immunofluorescence Staining

BV2 cells were seeded onto coverslips placed in 6-well plates at 1 × 10^4^ cells/well. Following 24 h of culture, treatment was applied as outlined above. Post-treatment, cells were fixed overnight at 4 °C using 4% paraformaldehyde and washed with PBS on the following day. Permeabilization was carried out with 0.3% Triton X-100 (P1080, Solarbio Science & Technology, Beijing, China) for 5 min at room temperature, followed by blocking in 10% goat serum (SL038, Solarbio Science & Technology, Beijing, China) for 1 h. Double immunofluorescence staining was subsequently performed. Cells were first labeled with CoraLite 594-conjugated anti-CD16/32 antibody (1:500, CL594-65057, Proteintech, Chicago, IL, USA) at 4 °C for an overnight period. Afterward, they were probed with anti-CD206 primary antibody (1:500, R015133, Epizyme Biomedical Technology, Shanghai, China) at 4 °C overnight, and subsequently with Alexa Fluor 488-conjugated goat anti-rabbit IgG secondary antibody (1:300, A-11034, Thermo Fisher Scientific, Waltham, MA, USA) for 1 h at room temperature protected from light. Following the staining procedure, coverslips were mounted onto glass slides using antifade mounting medium containing DAPI (S2110, Solarbio Science & Technology, China) and observed under a confocal fluorescence microscope. They were then semi-quantitatively analyzed using ImageJ software (v1.54d, National Institutes of Health, Bethesda, MD, USA).

### 4.15. Western Blot

As per the manufacturer’s instructions, the mouse hippocampus and BV2 cells were lysed using RIPA strong lysis buffer (PC101, Epizyme Biomedical Technology, Shanghai, China) containing protease and phosphatase inhibitors (GRF103, Epizyme Biomedical Technology, Shanghai, China). Following centrifugation, the supernatant protein content was measured by BCA assay and standardized across all samples. Protein samples were loaded in equal amounts and run on 7.5% SDS-PAGE gels (200 V, 35 min), followed by electroblotting onto 0.45 μm PVDF membranes (IPVH00010, Merck Millipore, Burlington, MA, USA) at 400 mA for 1 h. Membranes were then incubated in rapid blocking buffer (PS108P, Epizyme Biomedical Technology, Shanghai, China) for 30 min and probed with the following primary antibodies at 4 °C overnight: anti-HIF-1α (1:1000, WL01607, Wanlei Biotechnology, Shenyang, China), anti-mTOR (1:1000, 2972S, Cell Signaling Technology, Danvers, MA, USA), anti-p-mTOR (1:1000, 2971S, Cell Signaling Technology, Danvers, MA, USA), anti-HK2 (1:1000, R010880, Epizyme Biomedical Technology, Shanghai, China), anti-PKM2 (1:1000, GB11392, Service Biotechnology, Wuhan, China), anti-iNOS (1:1000, 52520, GenuIN Biotechnology, Hefei, China), anti-ARG-1 (1:2000, R014616, Epizyme Biomedical Technology, Shanghai, China), anti-SDHA (1:1000, S0B0929, Starter Biotechnology, Hangzhou, China), and anti-ATP5A (1:1000, S0B1054, Starter Biotechnology, Hangzhou, China). β-actin (1:8000, 20536-1-AP, Proteintech, Chicago, IL, USA) and tubulin (1:1000, 2128S, Cell Signaling Technology, Danvers, MA, USA) served as internal loading controls for hippocampal tissues and BV2 cells, respectively. The next day, after three washes with TBST, membranes received the matching secondary antibody (1:3000, 7074S, Cell Signaling Technology, Danvers, MA, USA) incubation for 1 h. After a further washing step, enhanced chemiluminescence (ECL) reagent (PK10003, Proteintech, Chicago, IL, USA) was applied to the target bands on the membranes, and protein bands were visualized and imaged using a gel imaging system (Bio-Rad Laboratories, Hercules, CA, USA). Densitometric analysis was performed in ImageJ. The signal of each target protein was divided by that of its internal reference to obtain relative expression values.

### 4.16. Statistical Analysis

Statistical analyses were conducted with SPSS (v26.0, IBM Corporation, Armonk, NY, USA), and results are expressed as mean ± standard deviation. The Shapiro–Wilk test was applied to assess data normality. For variables meeting the normality assumption, one-way ANOVA was performed, followed by Levene’s test to evaluate variance homogeneity. When variances were equal, Fisher’s LSD method was used for pairwise comparisons; otherwise, Tamhane’s T2 test was employed. For data that did not meet normality, the Kruskal–Wallis test with Dunn’s post hoc correction was utilized. In addition, for Western blot data of in vivo experiments, a randomized complete block design ANOVA was used. Results were shown as estimated marginal mean ± standard error derived from the model. A threshold of *p*-value < 0.05 was defined as statistically significant.

## 5. Conclusions

In conclusion, this study provides new evidence for the regulatory role of BWF in microglial metabolic reprogramming and immune phenotypic switching under DM conditions. BWF may drive microglial polarization away from a glycolysis-dependent pro-inflammatory profile toward an OXPHOS-dominated anti-inflammatory profile, an effect that is accompanied by the suppression of mTOR/HIF-1α pathway-mediated metabolic shift. These changes may collectively support the restoration of cellular energy metabolic homeostasis, alleviation of neuroinflammation, and improvement of cognitive function. These findings suggest BWF warrants further investigation as a potential therapeutic candidate for DACD.

## Figures and Tables

**Table 1:** The detailed detection data of components in BWF-CCSF.

No.	Type	Mode	RT (min)	Compound	*m*/*z*	Class	Source
1	Prototype	NEG	7.62	Chlorogenic acid	399.0929	Alcohols and polyols	Ginseng Radix, Polygalae Radix
2	Prototype	NEG	14.94	Ginsenoside Re	991.5489	Terpene glycosides	Ginseng Radix, Poria
3	Prototype	NEG	14.96	Ginsenoside Rg1	845.4908	Terpene glycosides	Ginseng Radix, Acori Tatarinowii Rhizoma, Poria
4	Prototype	NEG	16.53	Polygalasaponin XXIV	1235.5699	Terpene glycosides	Polygalae Radix
5	Prototype	NEG	18.45	Ginsenoside Rd	991.549	Terpene glycosides	Ginseng Radix, Poria
6	Metabolite	POS	7.75	Sinapic Acid	418.1343	Hydroxycinnamic acids and derivatives	Polygalae Radix
7	Prototype	POS	10.28	Sibiricaxanthone B	539.1395	1-benzopyrans	Polygalae Radix
8	Prototype	POS	10.86	Polygalaxanthone XI	569.1496	1-benzopyrans	Polygalae Radix
9	Prototype	POS	12.00	Cimifugin	307.1172	1-benzopyrans	Polygalae Radix
10	Metabolite	POS	13.94	5,7,4′-Trihydroxy-6-methoxyflavanone	479.1179	O-methylated flavonoids	Acori Tatarinowii Rhizoma
11	Prototype	NEG	16.29	3″O-Acetylplatycodin D	1265.5806	Terpene glycosides	Polygalae Radix
12	Metabolite	POS	16.66	26-Hydroxyporicoic acid DM	721.3804	Triterpenoids	Poria, Poria cum Radix Pini
13	Metabolite	POS	17.65	Ganolucidic acid B	696.3942	Triterpenoids	Poria, Poria cum Radix Pini
14	Prototype	NEG	17.81	Ginsenoside Rb2	1123.5909	Terpene glycosides	Ginseng Radix, Poria
15	Prototype	POS	22.04	Poricoic acid B	485.3256	Triterpenoids	Poria, Poria cum Radix Pini

Abbreviations: RT, retention time; NEG, negative ion mode; POS, positive ion mode.

## Data Availability

The original contributions presented in this study are included in the article/Supplementary Material. Further inquiries can be directed to the corresponding authors.

## Supplementary Materials

The following supporting information can be downloaded at: https://www.mdpi.com/article/10.3390/ph19071032/s1, Figure S1: The extraction ion current diagrams of 15 components in BWF-CCSF; Figure S2: Determination of the optimal non-cytotoxic concentration of BWF-CCSF for BV2 cell treatment; Table S1: Detection information of compounds in BWF aqueous extract; Table S2: Detection information of compounds in BWF-containing serum; Table S3: Detection information of compounds in BWF-CCSF.

## Abbreviations

The following abbreviations are used in this manuscript:

AD	Alzheimer’s disease
ARG-1	Arginase-1
ATP	Adenosine triphosphate
ATP5A	ATP synthase subunit alpha, mitochondrial
BWF	Buwang formula
BWF-CCSF	BWF- containing cerebrospinal fluid
DACD	Diabetes-associated cognitive dysfunction
DM	Diabetes mellitus
GO	Gene Ontology
HIF-1α	Hypoxia-inducible factor-1α
HK2	Hexokinase 2
iNOS	Inducible nitric oxide synthase
KEGG	Kyoto Encyclopedia of Genes and Genomes
mTOR	Mammalian target of rapamycin
OXPHOS	Oxidative phosphorylation
PKM2	Pyruvate kinase M2
PPI	Protein–protein interaction
SDHA	Succinate dehydrogenase [ubiquinone] flavoprotein subunit, mitochondrial
UHPLC-MS/MS	Ultra-high performance liquid chromatography-tandem mass spectrometry
