# Supplementary material for: Buwang Formula Regulates Microglial Metabolic Reprogramming and Modulates the mTOR/HIF-1α Pathway to Reduce Neuroinflammation in Diabetic Mice
Source: Pharmaceuticals (Basel). 2026 Jul 1;19(7):1032. doi: 10.3390/ph19071032 (PMC13415125; doi:10.3390/ph19071032)

1-Chlorogenic acid

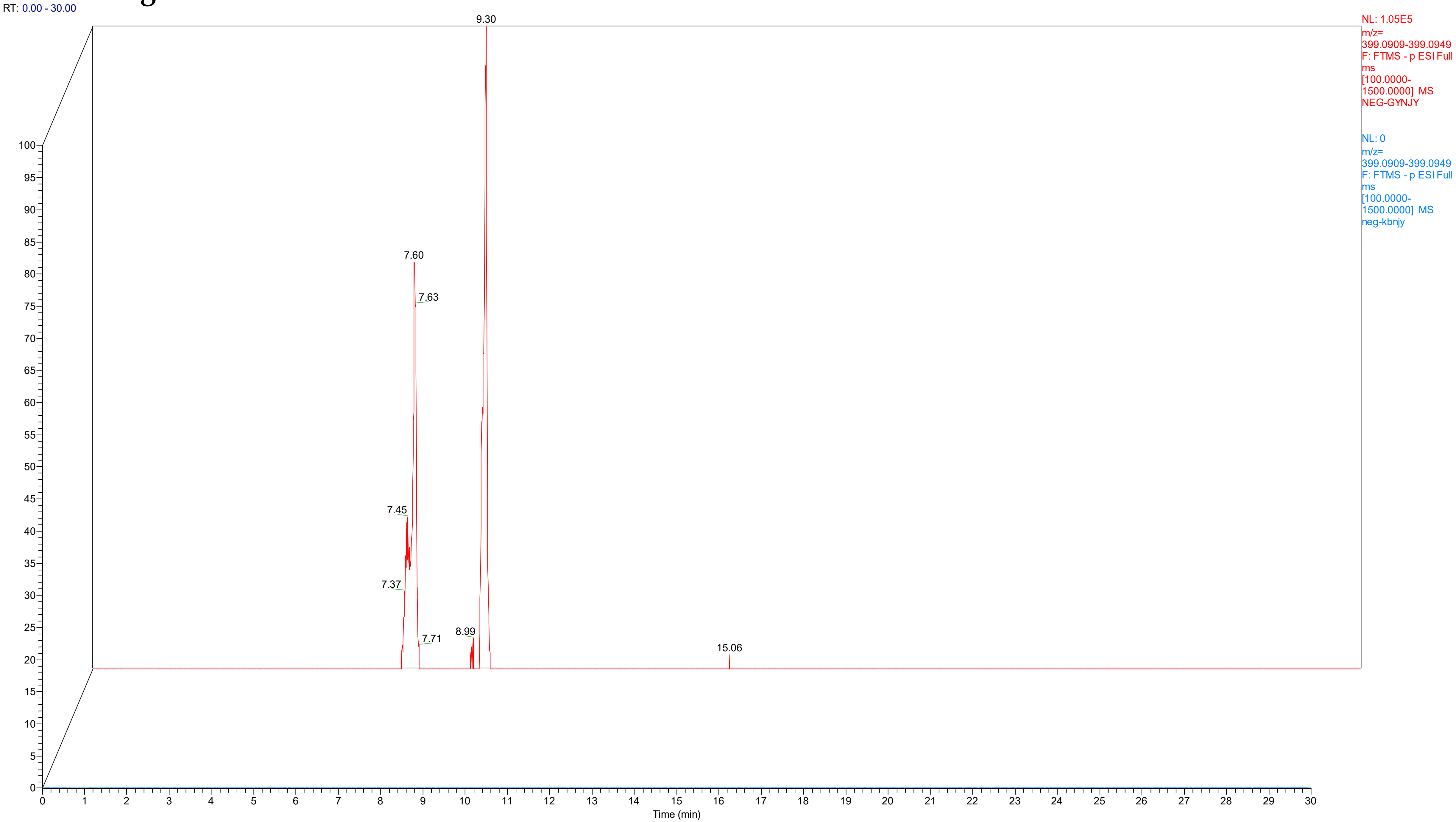

2- Ginsenoside Re

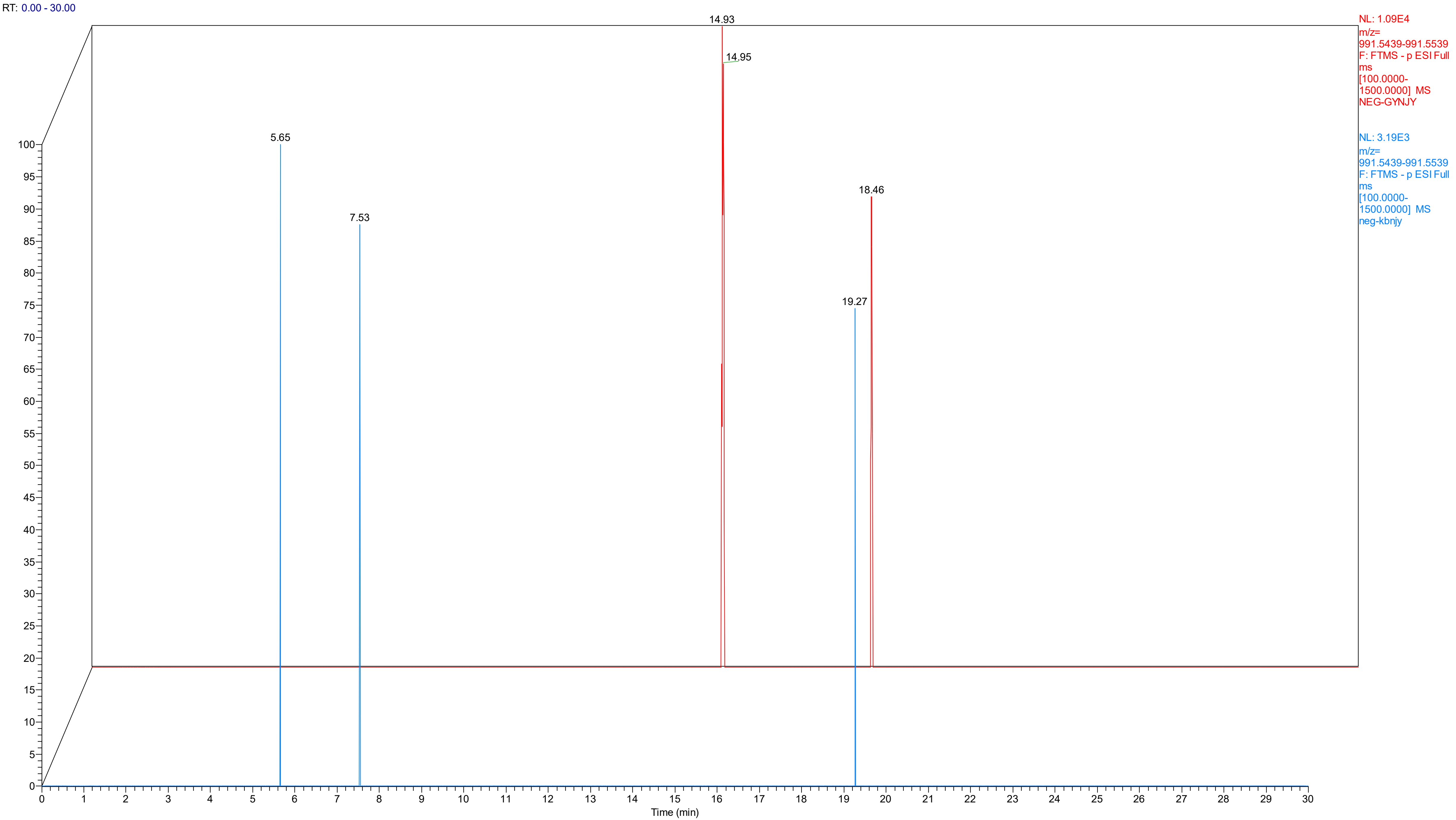

3- Ginsenoside Rg1

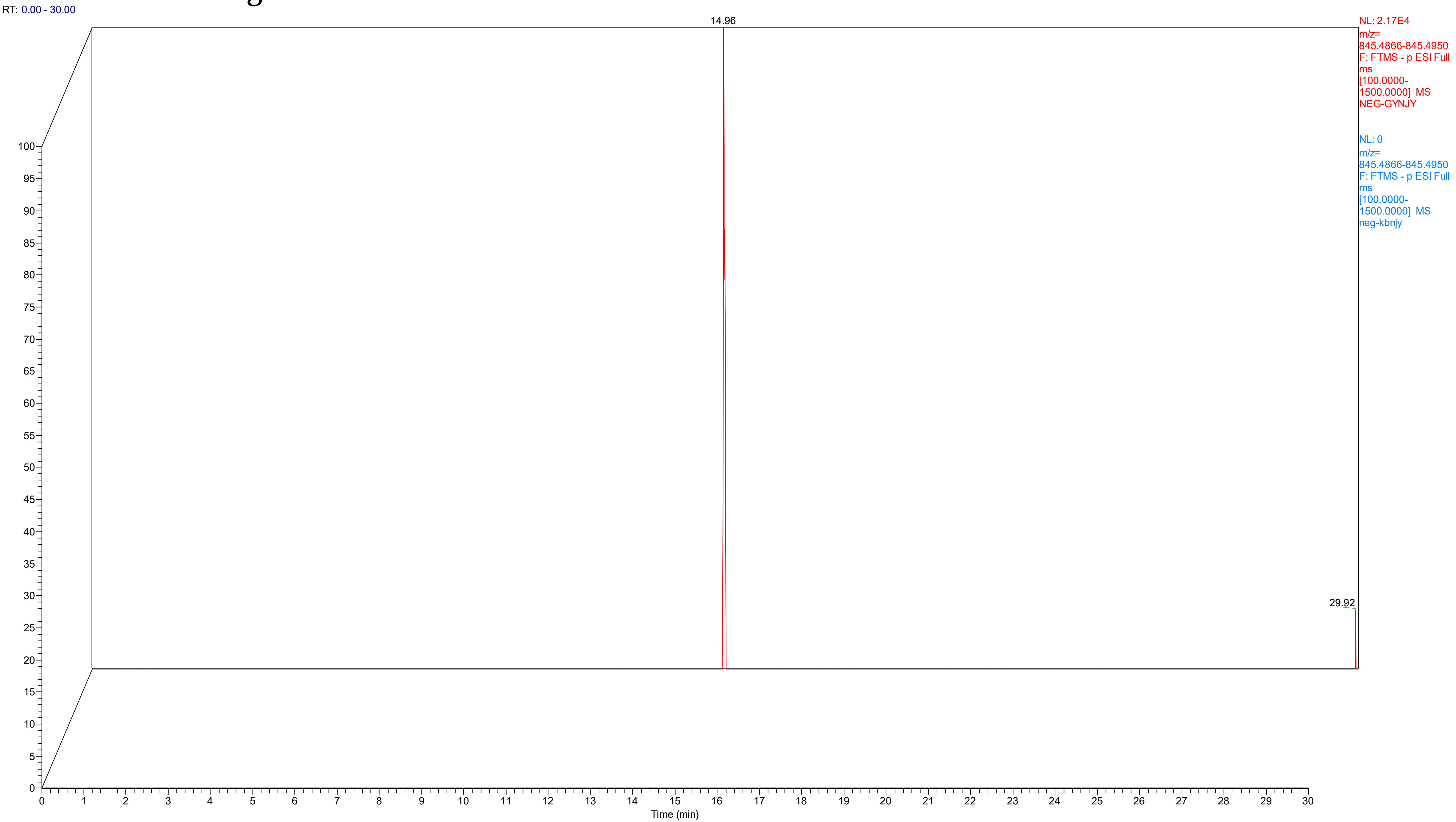

4- Polygalasaponin XXIV

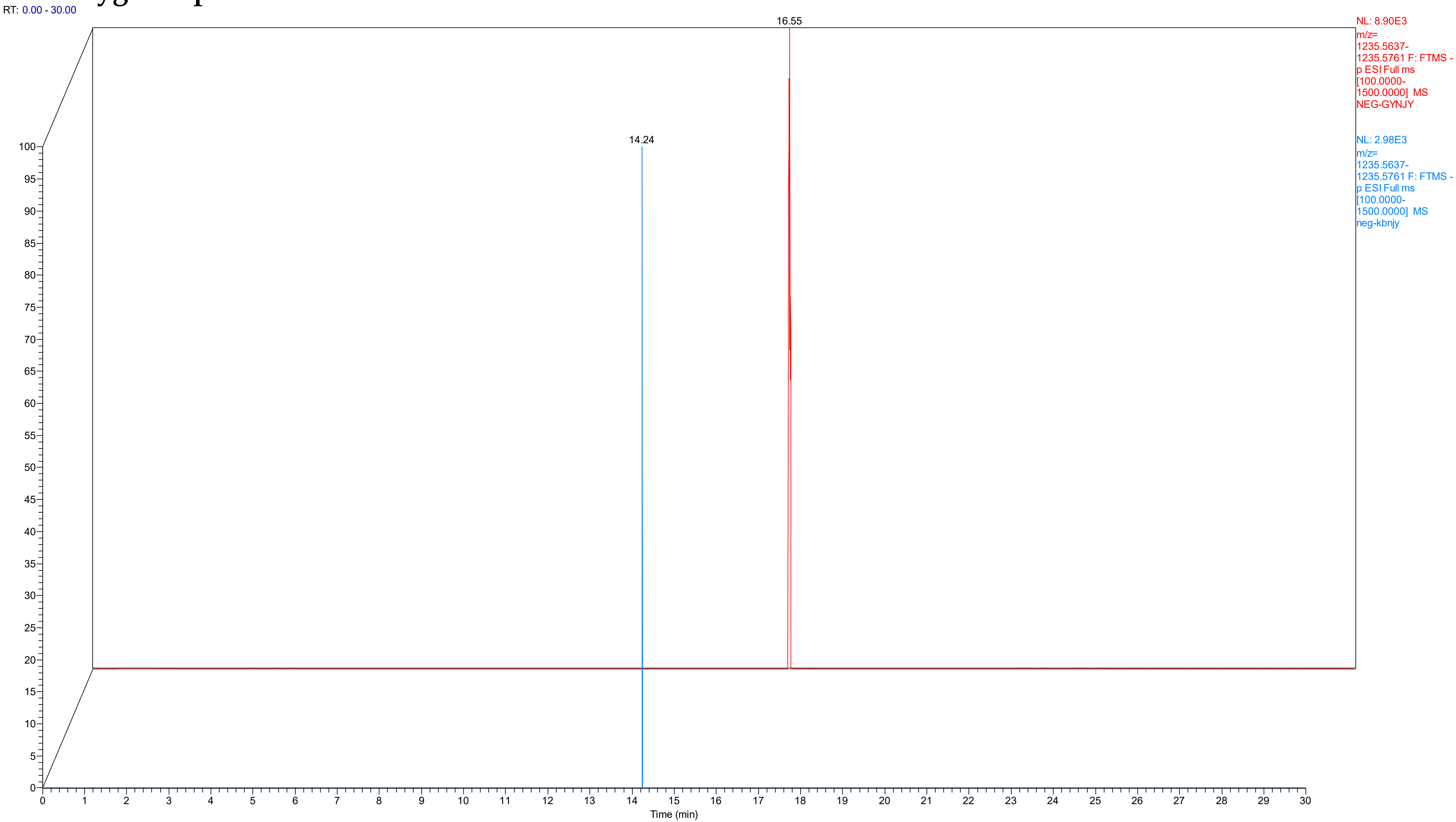

5- Ginsenoside Rd

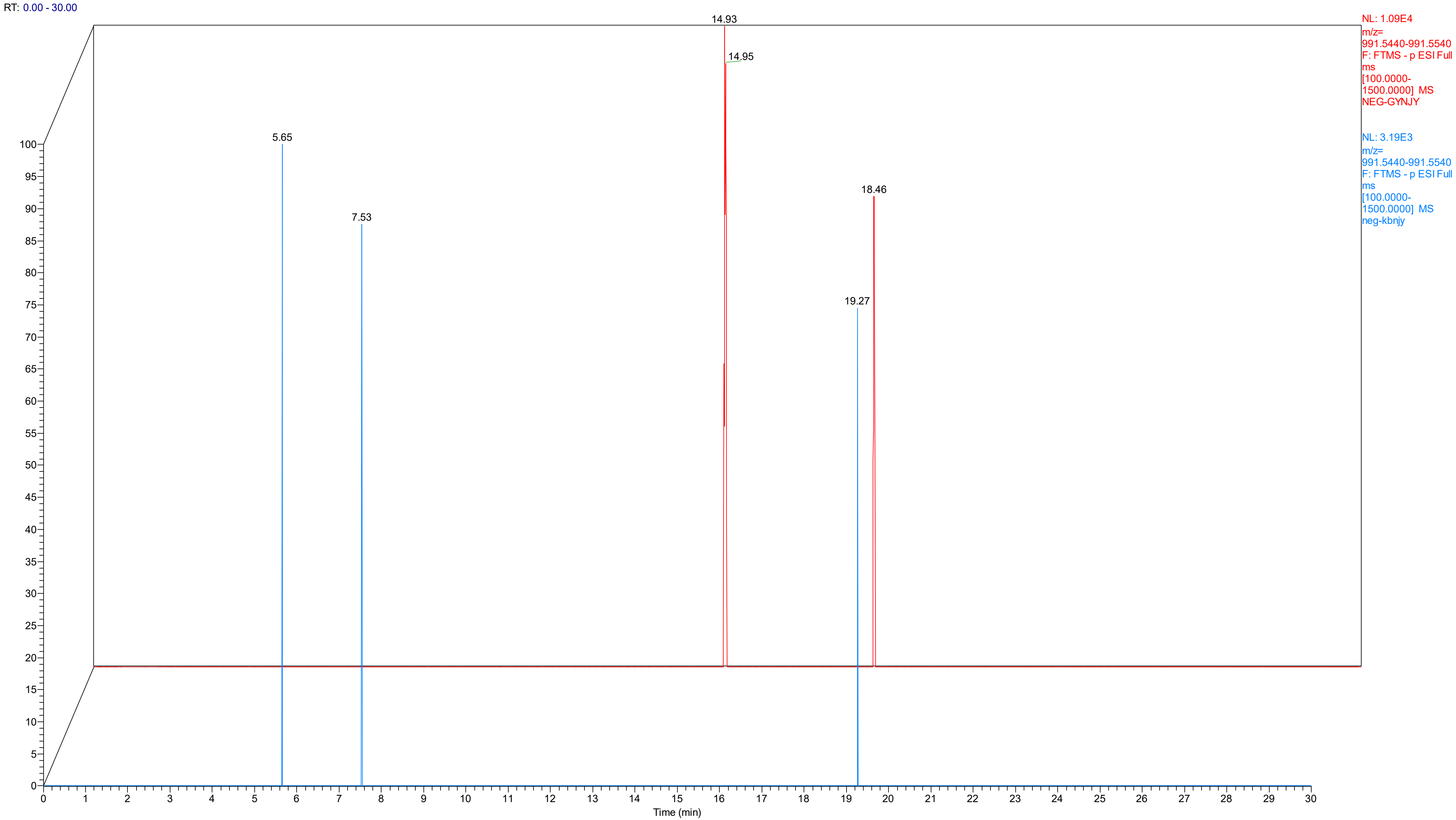

6- Sinapic Acid

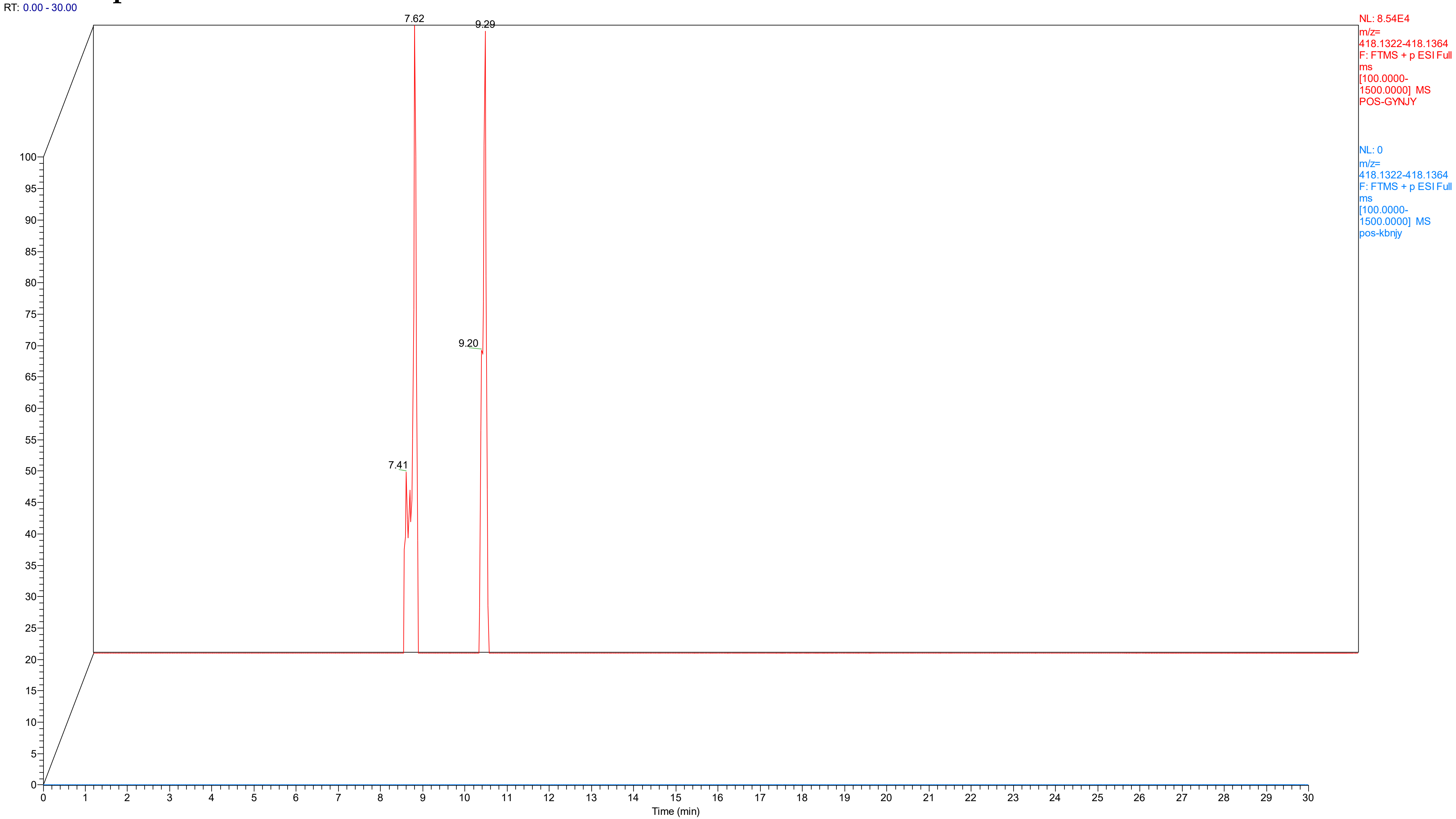

7- Sibiricaxanthone B

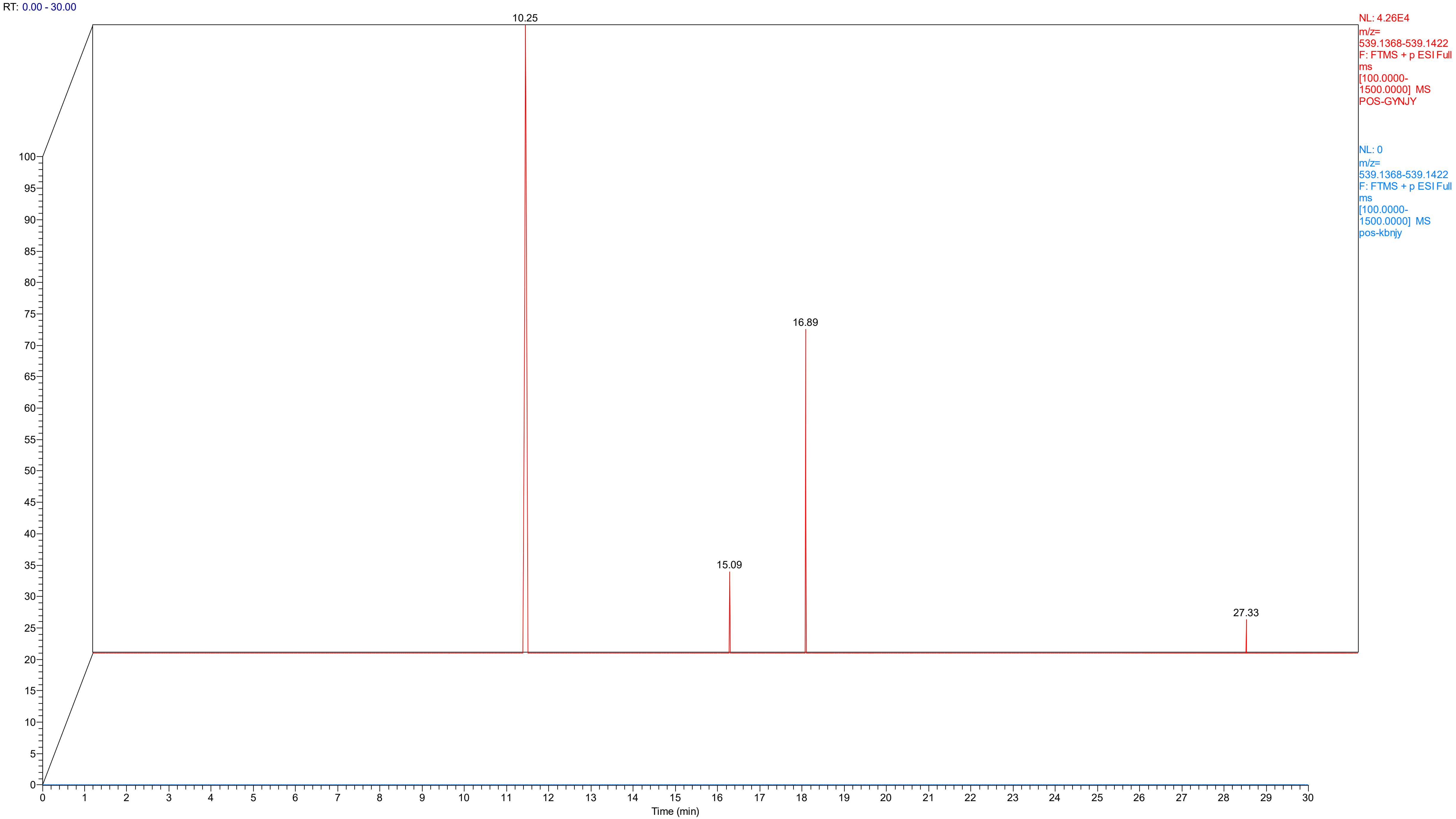

8- Polygalaxanthone XI

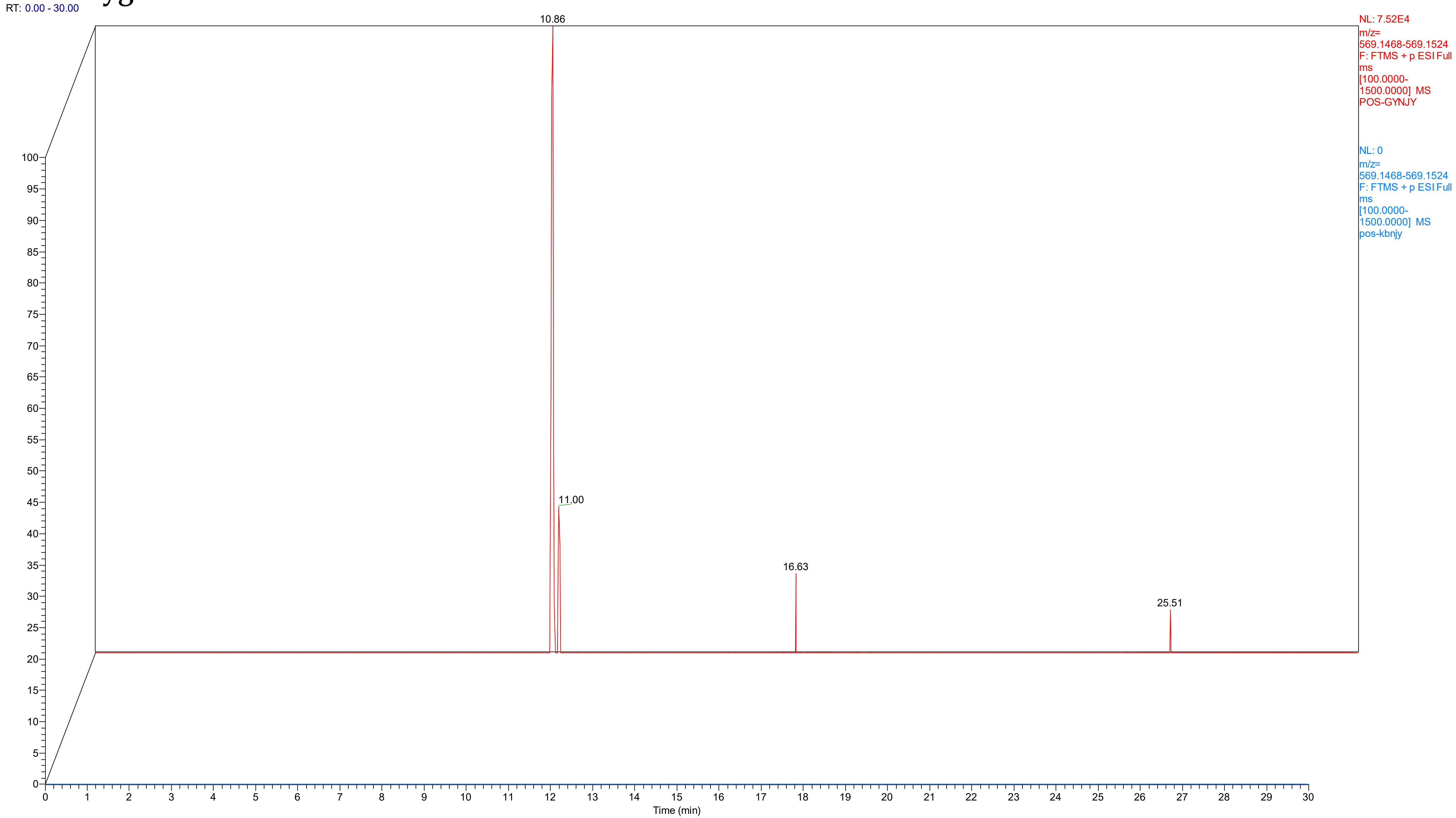

9- Cimifugin

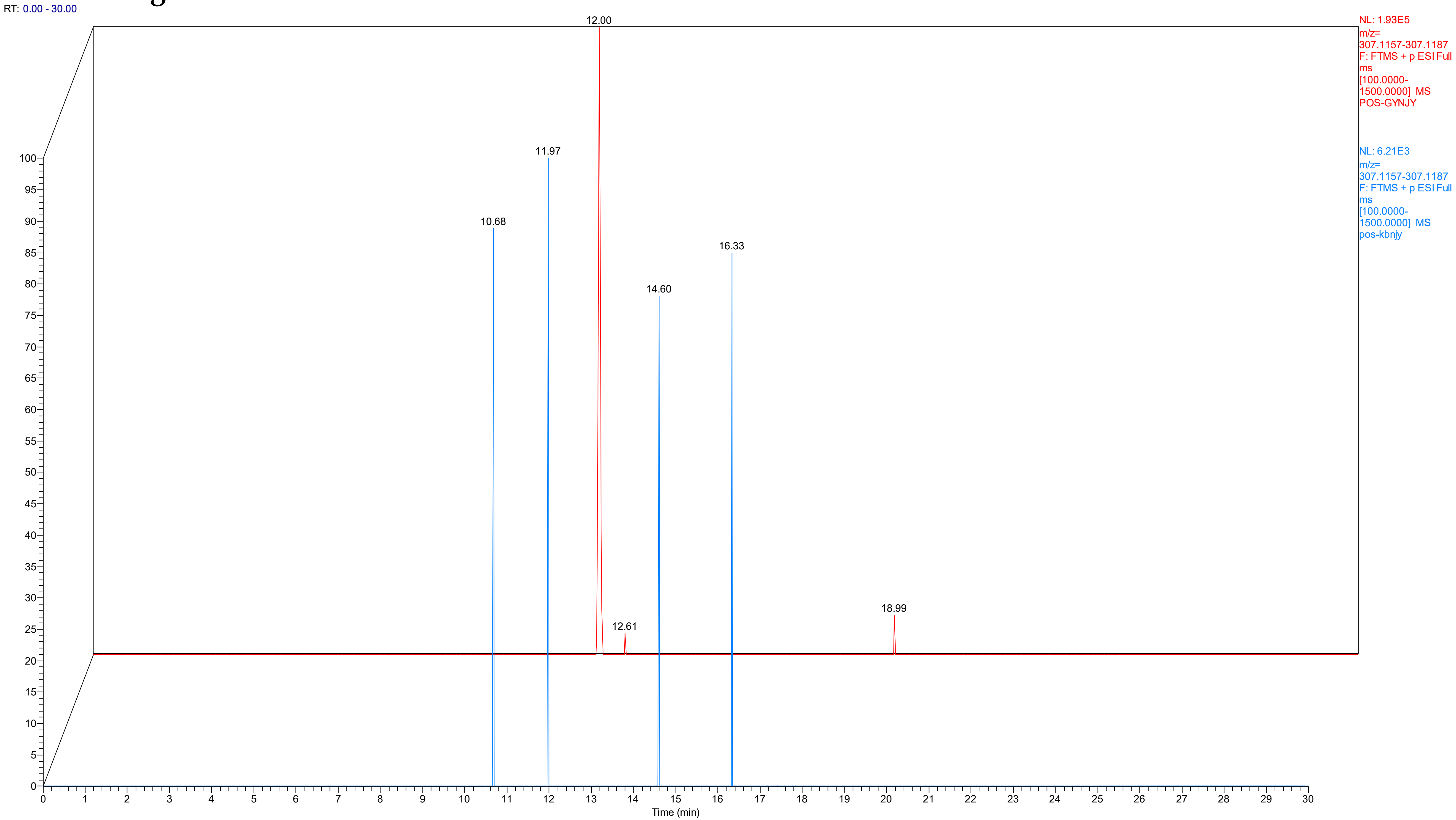
RT: 0.00 - 30.00
12.00
11.97
10.68
16.33
14.60
12.61
18.99
NL: 1.93E5
m/z= 307.1157-307.1187
F: FTMS + p ESI Full ms [100.0000-1500.0000] MS POS-GYNJY
NL: 6.21E3
m/z= 307.1157-307.1187
F: FTMS + p ESI Full ms [100.0000-1500.0000] MS pos-kbnjy
Time (min)

10- 5,7,4'-Trihydroxy-6-methoxyflavanone

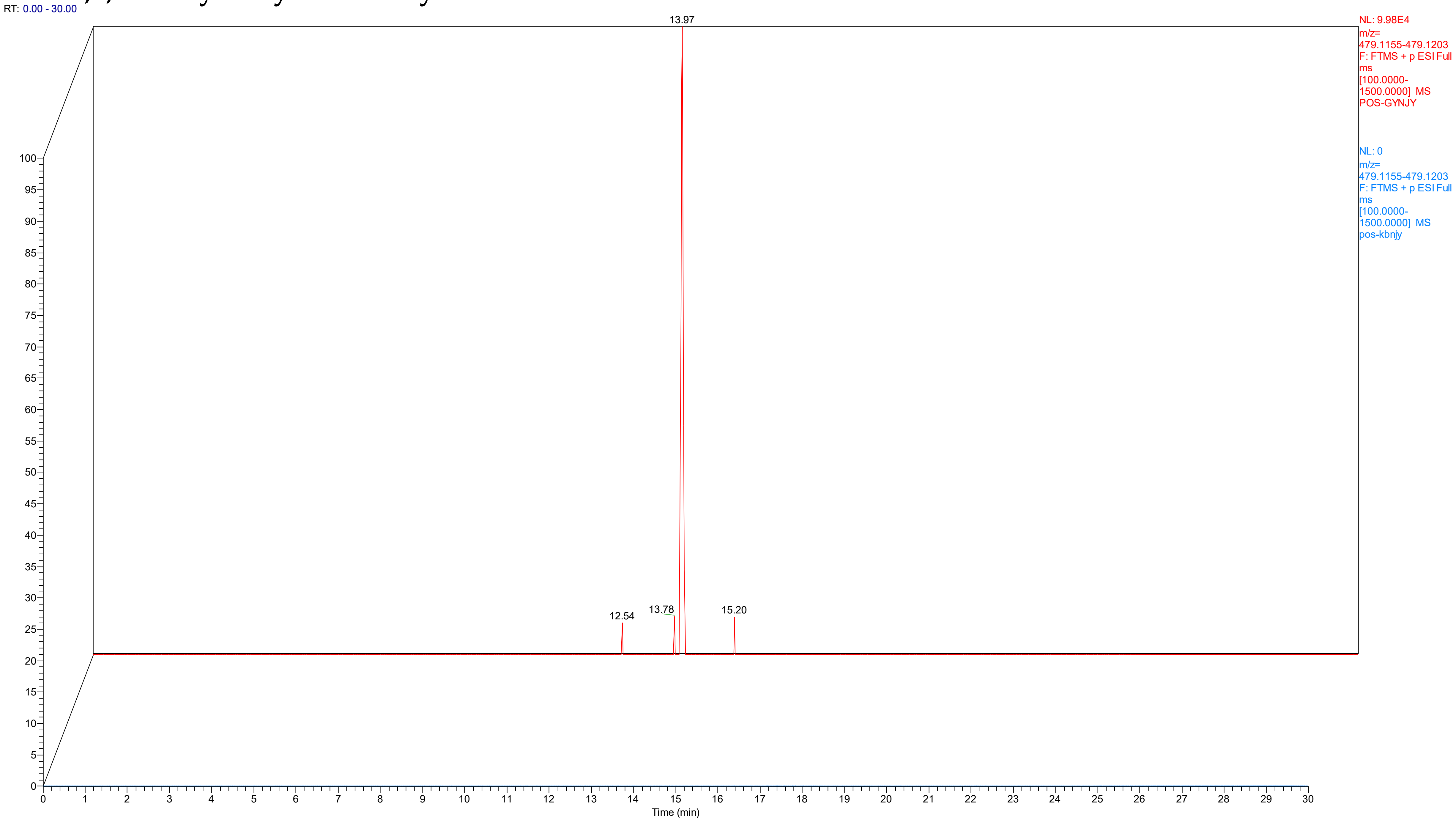

11- 3''O-Acetylplatycodin D

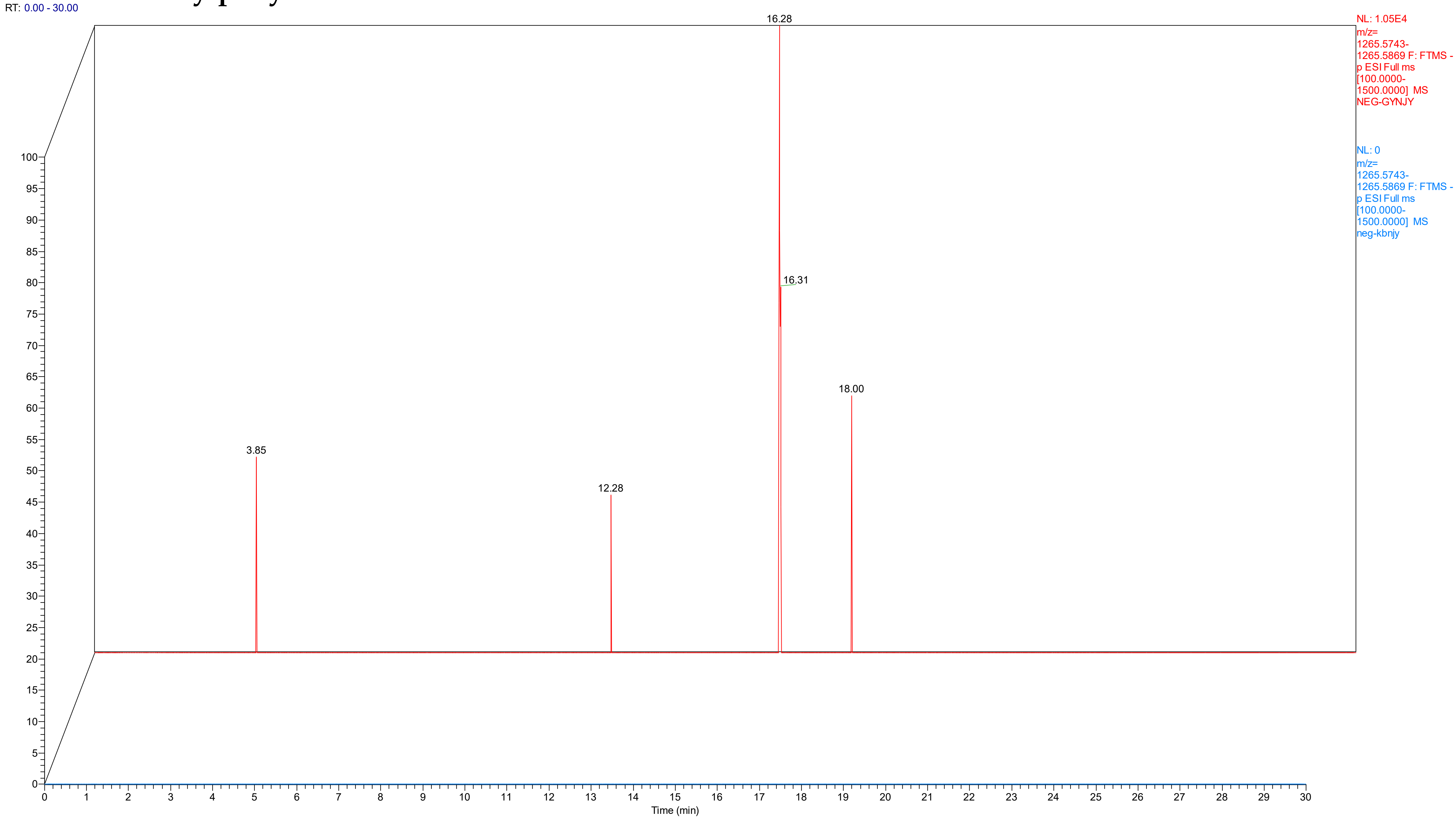

12- 26-Hydroxyporicoic acid DM

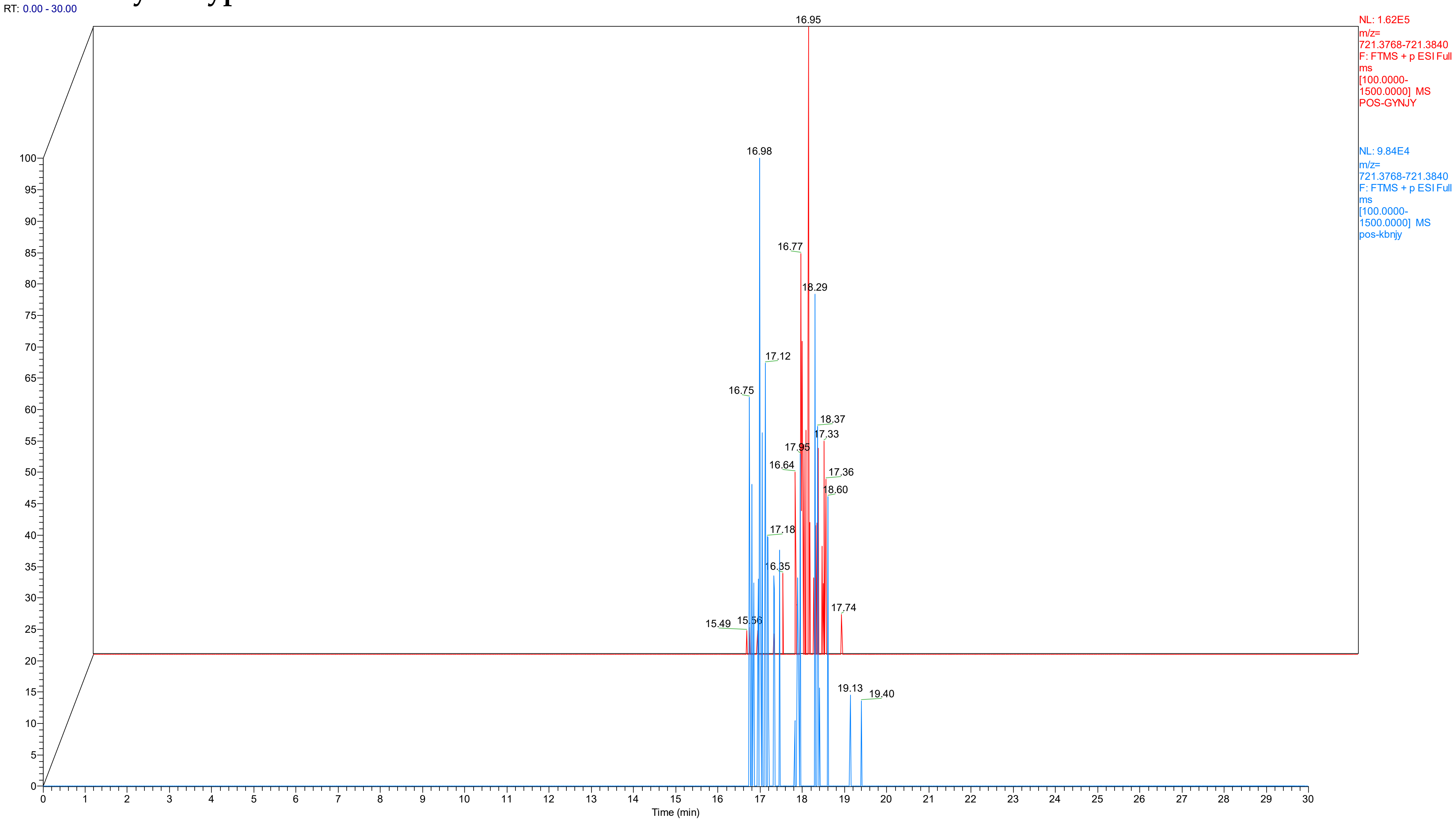

13- Ganolucidic acid B

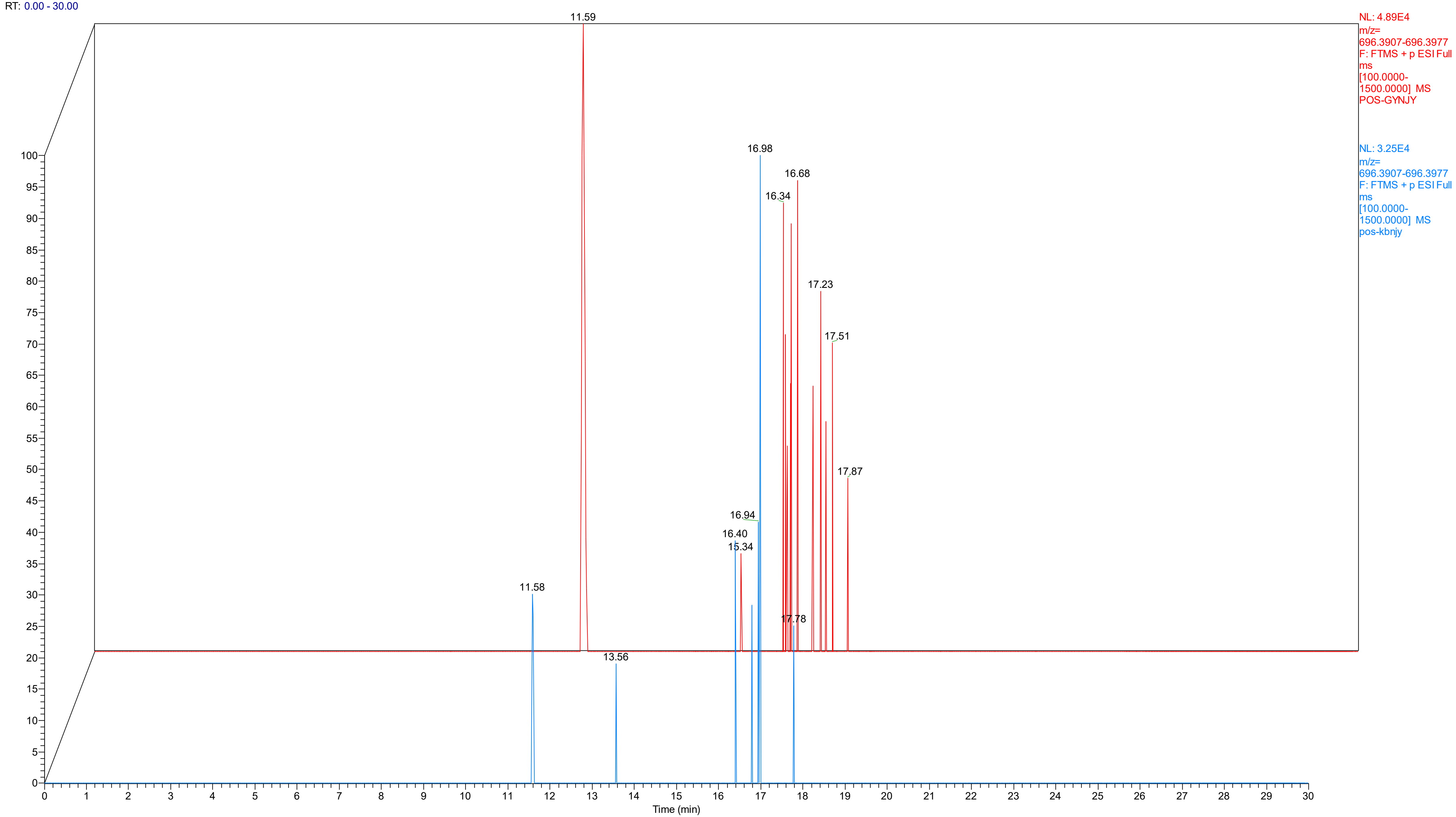

14- Ginsenoside Rb2

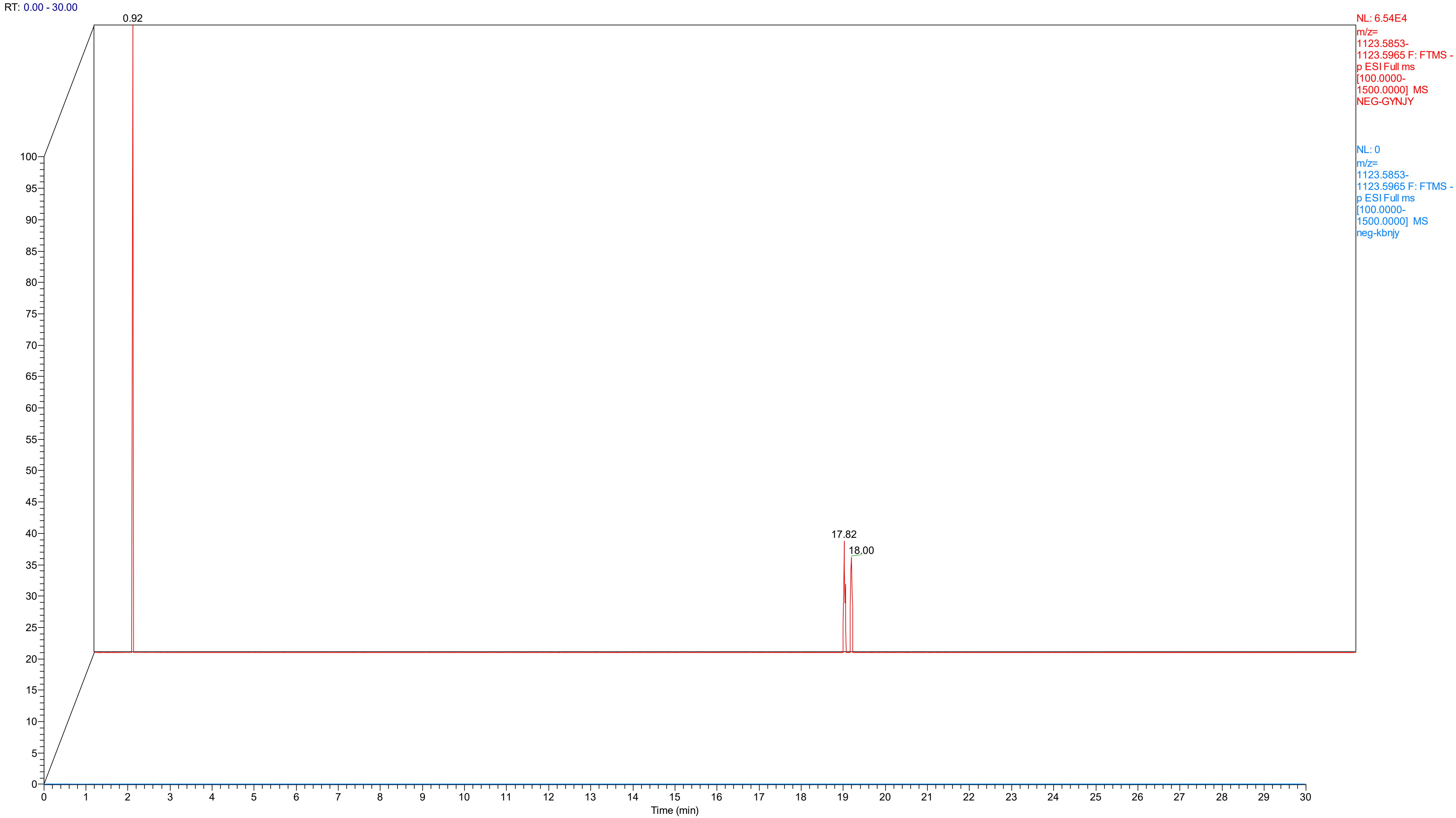

15- Poricoic acid B

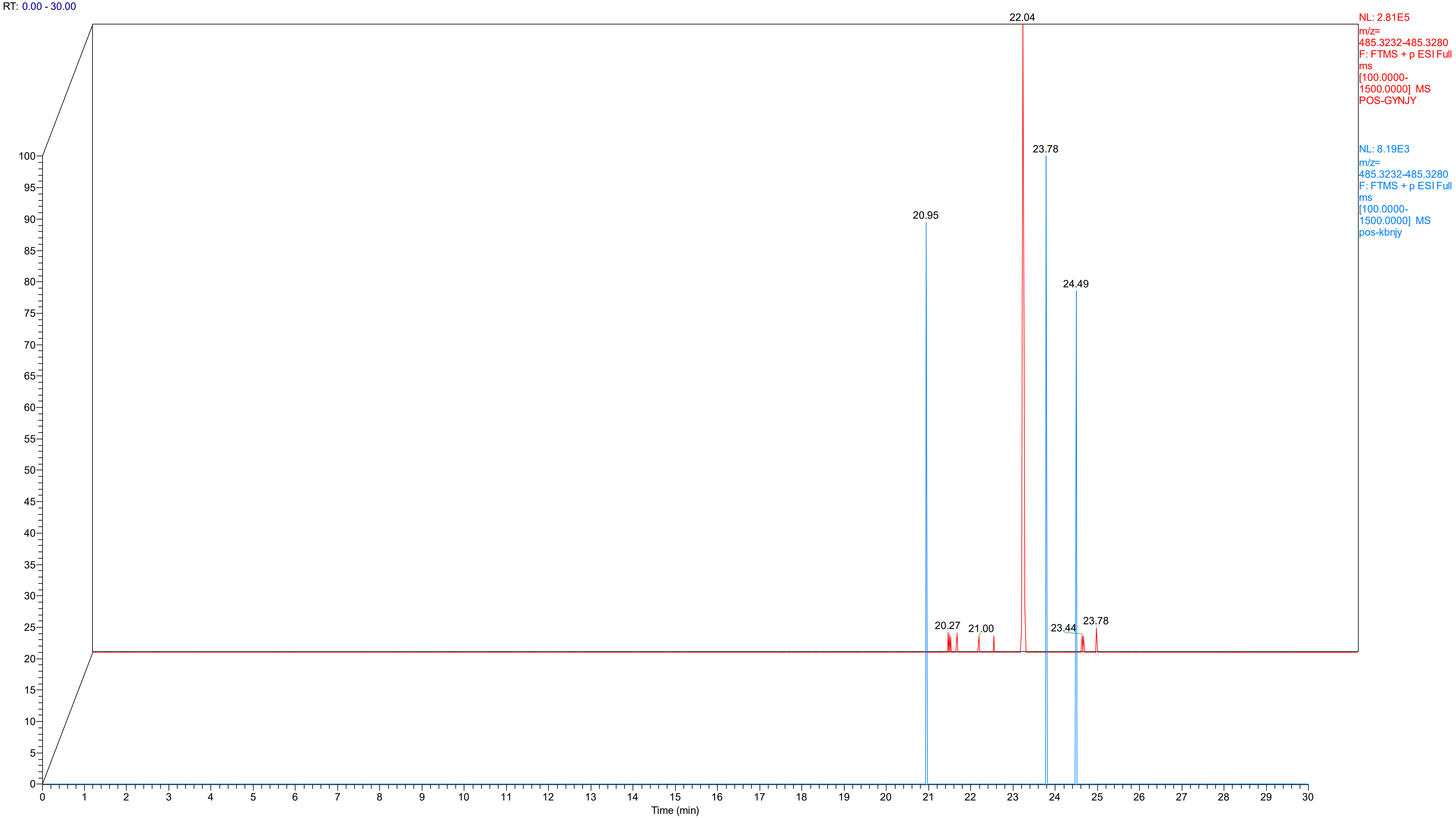

Supplement: Supplementary file 1 [file pharmaceuticals-19-01032-s001.zip › Figure S1. The extraction ion current diagrams of 15 components in BWF-CCSF.pdf]
