# Supplementary material for: Buwang Formula Regulates Microglial Metabolic Reprogramming and Modulates the mTOR/HIF-1α Pathway to Reduce Neuroinflammation in Diabetic Mice
Source: Pharmaceuticals (Basel). 2026 Jul 1;19(7):1032. doi: 10.3390/ph19071032 (PMC13415125; doi:10.3390/ph19071032)
Supplement: Supplementary file 1 [file pharmaceuticals-19-01032-s001.zip › Figure S2. Determination of the optimal non-cytotoxic concentration of BWF-CCSF for BV2 cell treatment.pdf]

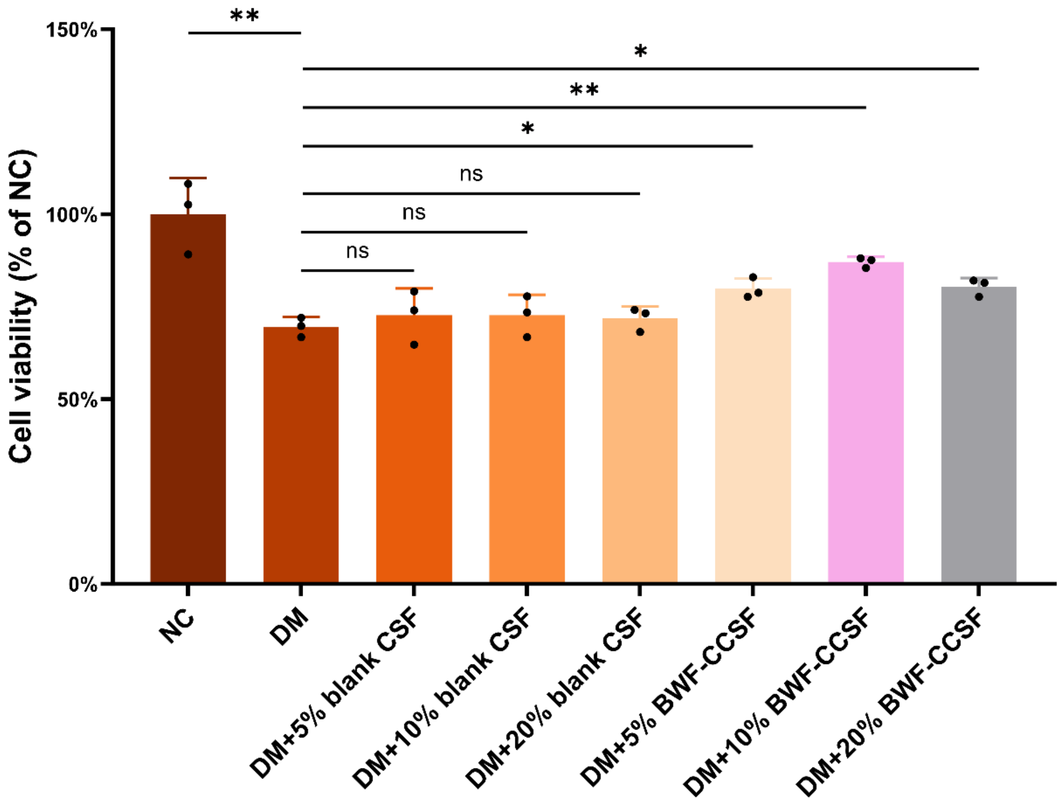

Figure S2. Determination of the optimal non-cytotoxic concentration of BWF-CCSF for BV2 cell treatment. BV2 cells were treated with different concentrations of blank CSF or BWF-CCSF for 24 h (n = 3). Blank CSF at 5%, 10%, and 20% did not significantly affect cell viability compared with the DM group ($p > 0.05$). In contrast, BWF-CCSF at all three concentrations significantly increased cell viability relative to the DM group ($p < 0.05$), with the 10% concentration producing the greatest improvement. Therefore, 10% BWF-CCSF was selected as the optimal concentration for subsequent experiments. * $p < 0.05$ and ** $p < 0.01$ vs. DM group.
